# Exploring Single-Atom Nanozymes Toward Environmental Pollutants: Monitoring and Control

**DOI:** 10.1007/s40820-025-01734-z

**Published:** 2025-04-28

**Authors:** Guojian Wu, Si Li, Linpin Luo, Yuechun Li, Wentao Zhang, Heng Wang, Sha Liu, Chenxing Du, Jianlong Wang, Jie Cheng, Yongning Wu, Yizhong Shen

**Affiliations:** 1 Engineering Research Center of Bio-Process, Ministry of Education, School of Food & Biological Engineering, Hefei University of Technology, Hefei, 230009 People’s Republic of China; 2 College of Food Science and Engineering, Northwest A & F University, Yangling, 712100 People’s Republic of China; 3 Institute of Quality Standards and Testing Technologies for Agro-Products, Chinese Academy of Agricultural Sciences, Beijing, 100081 People’s Republic of China; 4 NHC Key Lab of Food Safety Risk Assessment, Research Unit of Food Safety, China National Center for Food Safety Risk Assessment (CFSA), Chinese Academy of Medical Sciences (No. 2019RU014), Beijing, 100022 People’s Republic of China

**Keywords:** Single-atom nanozyme, Environmental health, Environmental pollutant, Monitoring, Control

## Abstract

As environmental pollutants pose a serious threat to socioeconomic and environmental health, the development of simple, efficient, accurate and cost-effective methods for pollution monitoring and control remains a major challenge, but it is an unavoidable issue. In the past decade, the artificial nanozymes have been widely used for environmental pollutant monitoring and control, because of their low cost, high stability, easy mass production, etc. However, the conventional nanozyme technology faces significant challenges in terms of difficulty in regulating the exposed crystal surface, complex composition, low catalytic activity, etc. In contrast, the emerging single-atom nanozymes (SANs) have attracted much attention in the field of environmental monitoring and control, due to their multiple advantages of atomically dispersed active sites, high atom utilization efficiency, tunable coordination environment, etc. To date, the insufficient efforts have been made to comprehensively characterize the applications of SANs in the monitoring and control of environmental pollutants. Building on the recent advances in the field, this review systematically summarizes the main synthesis methods of SANs and highlights their advances in the monitoring and control of environmental pollutants. Finally, we critically evaluate the limitations and challenges of SANs, and provide the insights into their future prospects for the monitoring and control of environmental pollutants.

## Highlights

- Review of the state-of-the-art synthesis strategies for single-atom nanozymes.
- Analysis of the recent advances in single-atom nanozymes for monitoring and control of environmental pollutants.
- Challenges and perspectives of single-atom nanozymes in environmental pollutants monitoring and control.

## Graphical abstract

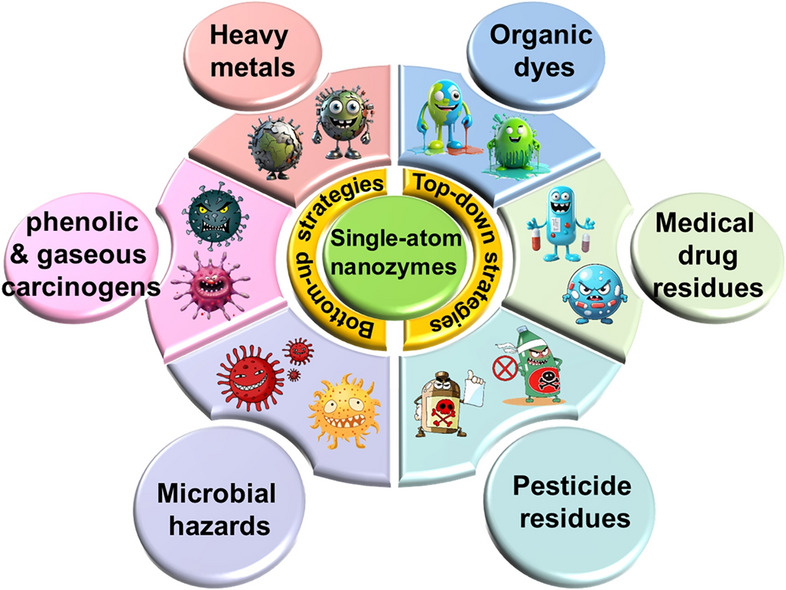

## Introduction

With the rapid development of the modern social economy, the problem of environmental pollution has become more and more increasingly prominent for enabling environmental health to be one of the biggest challenges faced by human beings [1]. According to statistics, there are many kinds of environmental pollutants, such as phenolic & gaseous carcinogens, organic dyes, pesticide residues, medical drug residues, microbial hazards, and heavy metals. Among them, the typical carcinogens such as harmful phenols and gaseous substances could enter the human body through air, water, food chain, whose long-term exposure can lead to the serious adverse effects like cancer [2]. Organic dyes usually affect the levels of salinity and oxygen in water [3, 4]; the residual pesticides and medical drugs could pollute the ecosystem [5, 6], while the microbial hazards are capable of causing a series of biological diseases [7]. Moreover, the residual heavy metals such as mercury and lead are difficult to degrade, which may also cause the cardiovascular and neurological diseases [8]. Under this background, it is particularly important and urgent to strengthen the monitoring and control of environmental pollutants for safeguarding environmental and public health.

Recently, as an emerging nanotechnology, artificial nanozymes of all kinds have been widely engineered in the monitoring and control of environmental pollutants with good results, owing to their features of low cost, high stability, easy mass production, etc. [7, 9, 10]. In spite of this, the conventional nanozyme technology faces challenges such as difficulty in regulating the exposed crystal surface, complex composition, low catalytic activity ascribable to the low density of active sites, and difficulty in precise regulation. [11]. In contrast, SANs are new type of artificial nanozymes with the single metal atom as the active center, exhibiting the comparable catalytic activity to that of natural metalloenzymes [12]. It has many impressive advantages over conventional nanozymes, three of which are as follows: (1) The active sites of SANs are highly dispersed, which can increase the contact between the substrate and the active sites, achieving the efficient substrate reactions and high catalytic activity to accelerate the degradation reaction of organic pollutants in the environment and enhance the treatment efficiency [11]. (2) The outer electron cloud distribution of a single metal atom in SANs exhibits high discreteness for endowing it to precisely regulate the surrounding ligand environment. As a result, the electron transfer becomes more efficient, which is conducive to activating the substrates and being to form products during catalytic reactions [13, 14]. (3) The strong interaction between the metal atoms and the carrier allows the metal atoms to be stably dispersed on the carrier, preventing the aggregation and separation of active sites, thus ensuring the highly durable catalytic activity of SANs. This is beneficial for ensuring the reliability, stability and reproducibility of SANs-based methods in environmental monitoring and control of pollutants under different environmental conditions (*e.g.*, temperature, acidity, alkalinity, and humidity) [15]. Up to now, SANs have exhibited a great potential and achieved an impressive advance in the monitoring and controlling applications for environmental pollutants through the catalytic detection/degradation, adsorption, redox effects, etc. [9, 11]. However, the structural characterization of single-atom spatial distribution in SANs presents a significant challenge [12, 16]. Spherical aberration-corrected transmission electron microscopy (AC-TEM) and synchrotron X-ray absorption spectroscopy (XAS) characterizations enable atomic-scale determination of single-atom spatial distributions, coordination, and electronic states, and the in *situ* characterization techniques (*e.g.*, Raman, X-ray photoelectron spectroscopy) track real-time evolution of catalytic active sites [17]. Nevertheless, observing single atom-carrier interfaces and resolving active site heterogeneity remain challenging. In future, the theoretical calculations-assisted spatio-temporally resolved characterization techniques will promote the in-depth analysis of the catalytic mechanism of SANs and the precise design of their environmental applications [18].

**Fig. 1:**
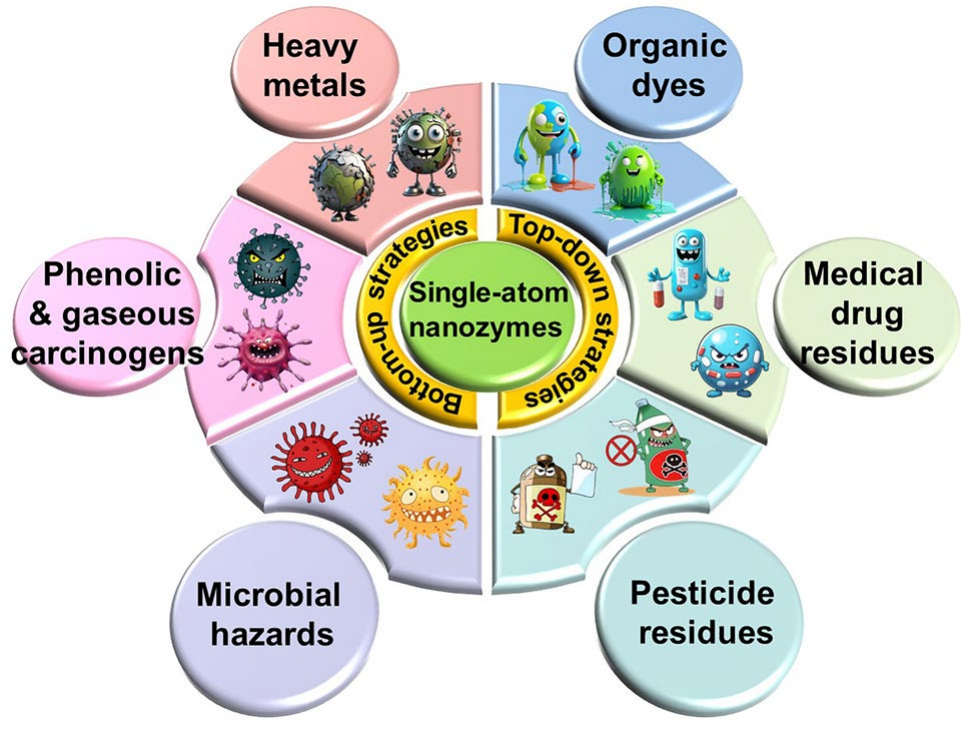
Summary of SANs for monitoring and control of different environmental pollutants (*e.g.*, phenolic & gaseous carcinogens, organic dyes, pesticide residues, medical drug residues, microbial hazards, and heavy metals)

Hence, this review concentrates on combing and analyzing the advances of SANs in the monitoring and control of environmental pollutants in detail (Fig. 1 and Table 1). The main synthetic strategies commonly involved in the synthesis of SANs are firstly summarized, including the “bottom-up” approaches of atomic layer deposition (ALD), mass-selective soft landing (MSSL), high-energy ball milling and photochemistry, as well as the “top-down” approaches of pyrolysis, gas-phase migration, chemical etching and sacrificial template method. Subsequently, the progress on the monitoring and control of SANs-based advanced methods to different environmental pollutants is also summarized, mainly including phenolic & gaseous carcinogens, organic dyes, pesticide residues, medical drug residues, microbial hazards, and heavy metals. On this basis, the application prospect and development direction of SANs in the monitoring and control of environmental pollutants are preliminarily foreseen and discussed. To the best of our knowledge, there has been insufficient effort to comprehensively characterize the application of SANs to the monitoring and control of environmental pollutants so far. In this case, this review is timely in providing an in-depth discussion and analysis on the advances of SANs in the efficient monitoring and control of environmental pollutants, so as to facilitate more efficient, accurate, reliable, economical, eco-friendly and commercially viable SANs-based methods for ultimately safeguarding environmental and public health.

**Table 1:** Comparison of the currently reported parameters of SANs for monitoring and control of different pollutants in environment-related matrices

Nanomaterials	Pollutants	LOD	Degradability (%)	Environment-related samples	*Refs*
Fe-CNG	HQ	0.025 µM	–	Lake & tap water	[71]
FeSA-NEPBC	BPS	–	90	Tap water & surface water	[72]
Fe-N-C	BPA	–	95	Tap water, Westlaker water & Xixi Wetland water	[73]
Fe/Mn-N-C	HQ	0.21 µM	92	–	[74]
Fe_1_@CN-20	4-chlorophenol, 2,6-dimeoxyphenol, 2,4-DP, catechol & phenol	3.3 µM 1.2 µM 1.3 µM 2.3 µM 2.6 µM	– 100 65 – –	–	[77]
Cu-Cys@COF-OMe	2,4-DP	–	70	Yongjiang river	[80]
SAFe-N-C & CDs	VA	0.9840 ppm (colorimetric) & 0.0838 ppm (fluorescent)	–	Pork &, beef, lamb & chicken	[83]
Pt_1_/ZnO	TEA	100.0 ppb	–	Air	[84]
Au_1_/In_2_O_3_	CO	10.0 ppm	–	Air	[85]
SnO_2_/Rh	formaldehyde	55.0 ppb	–	Air	[32]
Cu SA/WO_2.72_	toluene	10.0 ppb	–	Air	[86]
FeN_x_/g-C_3_N_4_	MB, RhB & MO	–	100	Drain water	[94]
Fe/g-C_3_N_4_	MO	–	95	–	[95]
ECN@FePc-600-2	RhB	–	99	–	[96]
Fe-N/FeS@C	MB	–	97.0	–	[92]
Fe-N-C/CNx	MG	–	92.1	–	[99]
FeBi-NC	RhB	–	100	–	[100]
Fe-SAs@FNC	PPD	0.07 µM	–	Hair dye	[101]
ZnBNC	PPD	0.1 µM	–	Hair dye	[102]
Mn-SAzyme	MB	–	90	Tap water & lake water	[103]
3DOM Fe-N-C	RhB	–	94	–	[104]
SACe-N-C	omethoate, methamidophos, carbofuran, carbosulfan	55.83 ng mL^−1^ 71.51 ng mL^−1^ 81.81 ng mL^−1^ 74.98 ng mL^−1^	–	Broccoli, ginger, rape & cabbage	[108]
Fe-N/C	chlorpyrifos	2.11 ng mL^−1^	–	Soil	[109]
Fe SAs/N_5_-pC-4	OPs	0.0006 μg mL^−1^	–	Lake water & tap water	[111]
Fe-N-C	acetamiprid	16.9 nM	–	Tap water	[112]
SA-CoN_3_	glyphosate	0.66 µM	–	Lake water, apples, pears, peaches & grapes	[113]
Fe-N/C	OPs	0.4177 nM	–	Cabbage, cucumber, fruit	[114]
Ir(III)/GO	PIB	3.31 nM	–	Pakchoi & apple	[115]
SA-Fe-NZ	OPs	3.55 fM	–	Vegetables	[118]
FeSAN	AMP	3.23 μM	–	–	[124]
Fe-N-C	acetaminophen		100	Seawater	[126]
Fe-SAC	NPX	–	100	Wastewater	[127]
SA Co-N-C	CQP	–	95	Lake water, deionized water & tap water	[128]
Fe-N-PC	IBP	–	100	Tap water, river water & rainwater & seawater	[130]
SAFe-MCN	SMX	–	98.7	Water	[131]
SA-Cu/rGO	SMX, meropenem & sulfafurazole	–	99	Pharmaceutical effluent	[132]
Fe-ISAs@CN	SDZ	–	90	Water	[133]
Pd_1_/N-C	FZD	3.3 nM	–	Tap water & lake water	[134]
FeSA	CIP	–	94	–	[136]
Fe1-GO	TC	–	93.6	River water, rain water, tap water & pharmaceutical wastewater	[138]
Fe-g-CN	TC	–	90	Lake water, surface water & taping water	[140]
Fe-g-C_3_N_4_/Bi_2_WO_6_	TC	–	93.5	Lake water, tap water & deionized water	[141]
SA-Pt/g- C_3_N_4_-K	OTC	10.3 µg L^−1^	–	–	[143]
SMAO-MrGO-ED	CF & IBF	–	98.43 & 98.12	–	[145]
Co-pCN	OTC	–	72.9	Water	[146]
Co SAC	TC	–	96.3	Simulated wastewater	[147]
Cu-CNND	*E. coli* & *S. aureus*	–	99	–	[156]
Fe-N-C	*E. coli* & *S. aureus*	–	82.1 & 88.1	–	[159]
Mn SACs	*E. coli* & *S. aureus*	–	98	–	[160]
SAF NCs	*E. coli* & *S. aureus*		82.6 & 94.38	–	[161]
Fe-N-C	AFB_1_	3.3 pg mL^−1^	–	Peanut	[166]
Fe-N-C	AFB_1_ & FB_1_	2.8 & 13.9 pg mL^−1^	–	Maize	[167]
Fe-N-C SAE	Hg^2+^	1.0 nM	–	Drinking water	[175]
Fe-N/S-C	Hg^2+^	0.17 nM	–	Tap water, lake water & sea water	[176]
SACe-N-C	Al^3+^	22.89 ng mL^−1^	–	Tofu & tofu skin products	[177]
SACe-N-C	Fe^3+^ & Cr^6+^	34.72 ng mL^−1^ & 93.65 ng mL^−1^	–	Drinking water, tap water, spinach & wheat	[178]
SA-Fe/NG	Cr^6+^	3.0 nM	–	Tap water & tuna	[179]

Limit of detection (LOD, calculated as 3σ/k) indicates sensitivity, where lower values denote higher sensitivity. Degradability is defined as the percentage of pollutants that are broken down and destroyed under certain conditions

Fe-CNG: single-atom Fe on *N*-doped graphene nanosheets; HQ: hydroquinone; BPS: 4,4-sulfonyl diphenol; FeSA-NEPBC: a single-atom iron catalyst with Fe-N_x_ configuration; Fe-N-C: Fe on nitrogen-doped porous carbon substrates; BPA: bisphenol A; Fe/Mn-N-C: manganese-amplified Fe-N-doped carbon nanozymes; Fe_1_@CN-20: iron single-atom anchored *N*-doped carbon material; Cu-Cys@COF-OMe: laccase-nanozyme supported on mesoporous COF-OMe; 2,4-DP: 2,4-dichlorophenol; SAFe-N-C&CDs: a single-atom iron nanozyme and carbon quantum dots; VA: volatile amines; TEA: triethylamine; SnO_2_/Rh: Rh-sensitized SnO_2_ nanoparticles; FeN_x_/g-C_3_N_4_: Fe sites embedded in graphitic carbon nitride; ECN@FePc-600-2: synthesize Fe single atom in situ growth on exfoliated graphitic carbon nitride; RhB: rhodamine B; MO: methyl orange; MB: methylene blue; Fe-N-C/CNx: Fe-N-C monoatomic catalyst/carbon nitride; MG: Malachite green; FeBi-NC: Fe-Bi bimetallic MOF-derived carbon supported Fe-N_4_ and Bi-N_4_ dual-site SAzyme; Fe-SAs@FNC: single atom based on single Fe atoms on fluorine-doped ultrathin carbon nanosheets; PPD: *p*-phenylenediamine; ZnBNC: B-doped Zn-N-C; Mn-SAzyme: single-atom nanozymes centered on Mn with *N*, *P*, and *S* doped; SACe-N-C: single-atom Ce-N-C nanozyme; Fe-N/C: iron single atom nanozymes; OPs: organophosphorus pesticides; Fe SAs/N_5_-pC-4: SAC with an Fe-N_5_ active center confined by hierarchically porous carbon nano frames; SA-CoN_3_: unsaturated cobalt-nitrogen sites doped within porous carbon; Ir(III)/GO: high-loading iridium on graphene oxide nanosheets; PIB: pirimicarb; SA-Fe-NZ: single-atom iron nanozyme; AMP: acetaminophen; FeSAN: Fe single-atom nanozymes; NPX: naproxen; SA Co-N-C: biochar-based SANs with cobalt single atoms; CQP: chloroquine phosphate; Fe-N-PC: porous iron/nitrogen-doped carbons; IBP: ibuprofen; SMX: sulfamethoxazole; SAFe-MCN: single atom Fe-dispersed g-C_3_N_4_ nanosheets; SA-Cu/rGO: the single-atom copper sites embedded in reduced graphene oxide; Fe-ISAs@CN: single-atom iron fixed on nitrogen-doped porous carbon; SDZ: sulfadiazine; FZD: furazolidone; Pd_1_/N-C: single-atom palladium anchoring in *N*-doped carbon; CIP: ciprofloxacin; FeSA: Fe single atom; Fe1-GO: FeP and Fe single-atom graphene oxide nanocomposite; Fe-g-CN: Fe single atoms and Fe clusters anchored to the g-C_3_N_4_ skeleton; Fe-g-C_3_N_4_/Bi_2_WO_6_: Z-scheme heterojunction photocatalysts of Fe-g-C_3_N_4_ and Bi_2_WO_6_; SA-Pt/g-C_3_N_4_-K: a SAzyme based on Pt single atoms midriffed carbon nitride nanorod; CF: ciprofloxacin; IBF: ibuprofen; SMAO-MrGO-ED: single metal atom oxide anchored Fe_3_O_4_-ED-rGO; OTC: oxytetracycline; Co-pCN: single-atom cobalt in polymeric carbon nitride; Cu-CNND: C_3_N_4_ nanodot-loaded single Cu atom nanozyme; *E. coli*: *Escherichia coli*; *S. aureus*: *Staphylococcus aureus*; Mn SACs: spherical mesoporous manganese single atom catalysts; SAF NCs: nano catalysts with single iron atoms anchored in nitrogen-doped amorphous carbon; AFB_1_: aflatoxin B_1_; Fe-N-C SAE: a uniform dodecahedral shaped *N*-doped carbon decorated by single Fe site enzyme; Fe-N/S-C: nitrogen and sulfur coordinated Fe-N/S-C SAzymes; SA-Fe/NG: Fe single-atom onto a single-layer of two-dimensional nitrogen-doped graphene

## Synthesis Approaches of SANs

**Fig. 2:**
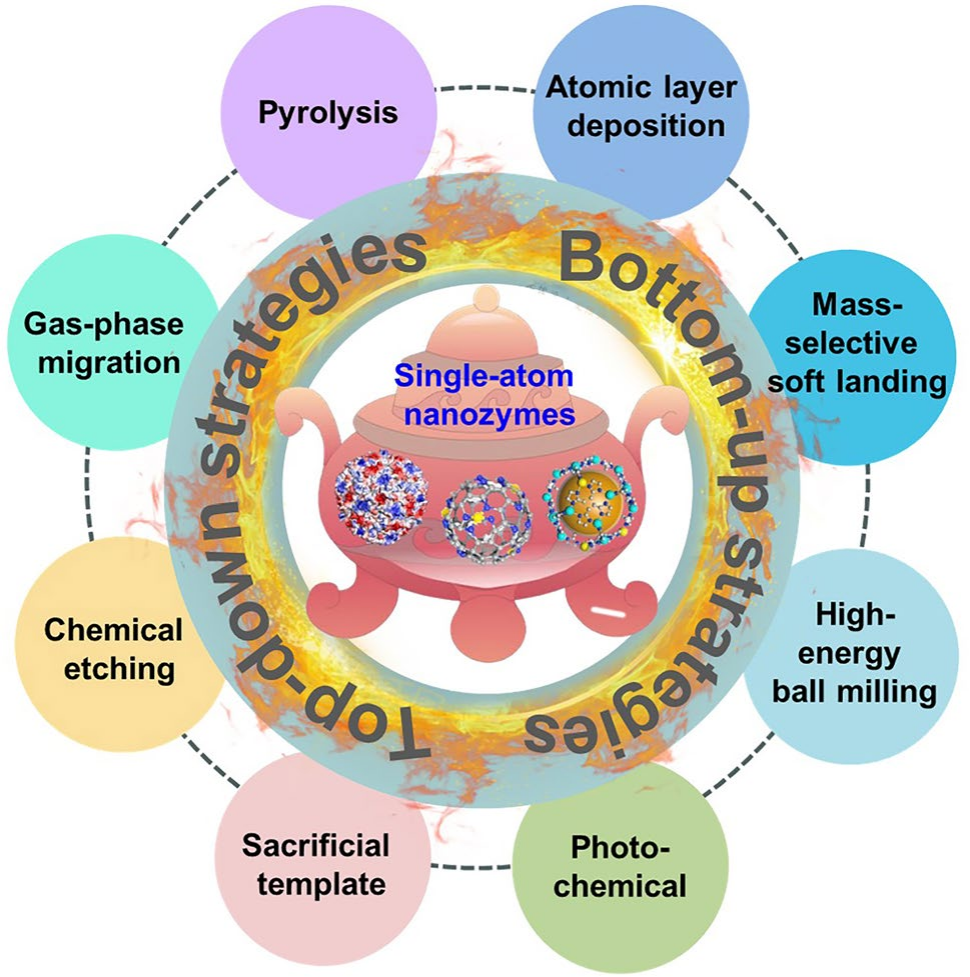
Schematic summary of the typical synthesis approaches for SANs

It is well-known that how to obtain and stabilize the isolated atomic sites is one of the main challenges in SANs synthesis at present. In general, when the size of the metal decreases to the atomic scale, some unsaturated coordination sites are formed, and isolated metal atoms tend to aggregate into nanoclusters with high surface energy [16]. Therefore, it is essential to prevent the aggregation of metal atoms when SANs is synthesized [17, 18]. With these in mind, a series of synthesis approaches for SANs have here summarized to be basically divided into the mononuclear metal complexes-derived “bottom-up” strategy and the bulk metals or nanoparticles-derived “top-down” strategy by relying on the dimensionality of metal precursors and synthesis pathway orientation (Fig. 2), respectively [19].

### Synthesis of SANs with “Bottom-Up” Strategy

The “bottom-up” synthesis strategy involves constructing nanostructured materials through hierarchical assembly, beginning with molecular, atomic, or ionic building blocks that are first organized into larger cluster structures, which subsequently assemble into the desired nanomaterial architecture [19]. The synthesis of SANs using the “bottom-up” approach entails introducing atomically stable metal centers, such as metal ions, complexes, or even metal-organic frameworks (MOFs), into the carrier material through methods like maceration, electrostatic adsorption, and co-deposition. After post-treatments, the desired SANs can be obtained. To achieve the homogeneous dispersion of single atom and prevent theirs migration and aggregation into nanoparticles, controlling the loading of metal species is the most direct and effective method [20, 21]. In addition, through continuous optimization of support materials and synthetic protocols, the metal-carrier interaction-based migration techniques have also significantly solved the problem of aggregation of isolated atoms, thus providing diverse strategies for the synthesis of SANs substances with high stability and atomic loading [22]. So far, the “bottom-up” strategy based on metal complexes as precursors mainly include ALD [23, 24], MSSL [25, 26], high-energy ball milling [27, 28], and photochemical methods [29, 30].

Among these techniques, ALD operates through self-limiting surface reactions to achieve atomic-scale thin film growth. As a complement to traditional wet chemical catalyst preparation methods, ALD provides a fine-tuned technological approach to construct SANs catalyst with near-atomic precision for pollutant detection applications [31]. For example, Zhou et al*.* reported a formaldehyde gas sensor constructed based on single-atom Rh-sensitized SnO_2_ nanoparticles prepared by ALD. The atomic dispersion of Rh significantly enhanced gas adsorption capacity and interfacial charge transfer, resulting in a response to formaldehyde ranging from 20.0 to 36.3 ppm, with nearly 23-fold enhanced sensitivity over pure SnO_2_ [32]. In a word, ALD provides an ideal model catalyst for fundamental studies of SANs, making it possible to explore the effects of particle size, carrier surface properties, and inclusions on the surface of metal or alloy nanoparticles on the catalytic properties. This makes ALD an indispensable tool for investigating the synthesis and structural relationships of loaded SANs [33, 34].

Generally, MSSL entails vaporizing the metal precursor via high-frequency laser evaporation and subsequently employing the mass-selective capability of a quadrupole mass spectrometer to precisely deposit metal clusters with varying atomic compositions onto the carrier surface using the soft-landing technique [35]. In 2000, Abbet et al*.* used MSSL to deposit Pd_n_ clusters on surface-exposed MgO to catalyze acetylene trimerization for the preparation of benzene. They found the target product benzene can be obtained at 300 K for n = 1, which is about 130 K lower than the reaction temperature of other catalysts, indicating that single-atom Pd has high catalytic activity at low temperature [36]. However, this method is characterized by harsh experimental conditions and high cost, requiring ultra-high vacuum preparation conditions and harsh selection of metal precursors. Moreover, it suffers from low yield and limited applicability to carriers with high specific surface areas or mesoporous materials, requiring further optimization by researchers.

As another way, photochemistry represents an advanced processing technique that utilizes photon energy to dissociate gaseous precursors into atomic species for substrate deposition [37, 38]. By regulating the reaction conditions and optimizing the chemical properties of the substrate surface, this method can precisely control the deposition location of atoms, the morphology and crystal structure of SANs with high quality and purity [39, 40]. Although photochemical synthesis offers many advantages, it still faces some challenges. For instance, the substrate materials must exhibit exceptional light-harvesting efficiency while maintaining stringent spectral selectivity toward the excitation source, necessitating meticulous design of both reaction systems and illumination conditions [41]. Meanwhile, photochemical synthesis usually requires consideration of a variety of factors, including the intensity of light irradiation, irradiation time, reaction temperature, reaction time, etc*.* [40]. All of these factors directly influence the yield and selectivity of the reaction and therefore require thorough optimization.

In contrast to the aforementioned methods, mechanochemistry is an effective synthetic technique that utilizes mechanical energy to induce physical and chemical transformations of solid precursors and chemical reactions between different solid materials [42]. Thereinto, with the advantages of low cost, scalability and environmental friendliness, high-energy ball milling has been emerged as one of the most promising approaches to prepare SANs to degrade pollutants in environment [43]. This approach initiates chemical reactions at the interface of reactants at lower temperatures by repeatedly squeezing the material using hardened steel or oxide spheres [44]. For example, Tang et al*.* demonstrated this approach by preparing atomically dispersed Ni catalysts on g-C_3_N_4_ nanosheets through ball milling, which exhibited enhanced photocatalytic performance in CO_2_ reduction compared to conventional counterparts [45]. Compared with ALD and MSSL, the high-energy ball milling method is simple and efficient, without amplification effect, and can easily prepare kilogram catalysts with no change in catalyst structure.

Despite significant progress in “bottom-up” synthesis of SANs, several critical challenges persist. For example: (1) In wet-chemical routes, the synthesis process usually involves multi-step procedures encompassing metal precursor adsorption, solvent evaporation, and subsequent reduction/stabilization stages, creating complex operational workflows [11]. (2) A critical limitation in high-temperature processing of SANs arises from thermally driven migration and coalescence of adsorbed metal precursors on support materials, leading to nanoparticle formation through sintering mechanisms. This phenomenon results in compromised target nanoscale dimensions, necessitating meticulous parameter optimization to achieve precise nanostructural control [16]. (3) During the “bottom-up” synthesis process, defects generation is inevitable. These defects may affect the properties and stability of the materials, controlling the number and distribution of defects is an important challenge [46]. (4) “Bottom-up” synthesis methods typically require finely controlled conditions and expensive equipment, which limits their applications to large-scale production [47]. In future, combining the “bottom-up” synthesis strategy with modern automation and computer control technology to improve the preparation precision and efficiency and realize the intelligent production of SANs may provide new opportunities for the development of SANs.

### Synthesis of SANs with “Top-Down” Strategy

The “top-down” synthesis strategy for SANs involves the conversion of bulk or micro- or nanostructured metals into individual atoms stabilized on the carrier, which avoids the nanoparticles sintering at high temperatures in the “bottom-up” strategy. However, achieving the disruption of metal-to-metal bonding within the metal system necessitates specialized strategies and may present greater thermodynamic challenges compared to the “bottom-up” method [48, 49]. Common techniques employed in the “top-down” strategy include pyrolysis, gas-phase migration, chemical etching and the sacrificial template method [50–52].

Usually, pyrolysis is an important chemical reaction process in which different chemical substances can be obtained by breaking and reorganizing the molecules of a substance under high temperature conditions [53]. In SANs synthesis, this process enables atomic dispersion through optimized selection of metal-organic precursors, precise temperature/time regulation, and catalytic/solvent engineering [54]. A representative demonstration by Meng et al. achieved thermally stabilized Fe single-atom catalysts via controlled pyrolysis of polyaniline-incorporated Fe-porphyrin MOF (PANI@PCN-224(Fe)), yielding *N*-doped porous carbon matrices with atomic Fe sites (PANI@PCN-224(Fe)-900) [55]. Although pyrolysis is effective for building monometallic sites in SANs, there are still some problems such as poor biocompatibility due to the usually hydrophobic surface, uncontrollable particle size from high-temperature sintering, high energy consumption, etc., all of which warrant further investigation.

Different from above, vapor phase migration involves the evaporation of metals at elevated temperatures followed by deposition onto substrates with enhanced interfacial interactions, producing atomically dispersed SANs with excellent thermal stability properties [56]. For example, Li et al*.* conducted a simple experiment in which they mixed N, C, Fe NPs, and NH_4_Cl together as a precursor for pyrolysis. Under thermal activation, the decomposition of NH_4_Cl produces gaseous hydrogen chloride, which can corrode Fe nanoparticles, and the resulting FeCl_2_ can be easily introduced into the carrier by vapor diffusion and forms monoatomic Fe [57]. Compared to pyrolysis-based SANs synthesis, the vapor phase migration demonstrates improved cost-effectiveness and reduced energy requirements.

Additionally, chemical etching is defined as a process utilizing controlled chemical reactions to remove material from a substrate’s surface. Typically, the etching solution reacts with the material on the surface of the object to form a layer of dissolution products that gradually strips away the surface of the original material to form single atoms of the metal [58]. For instance, Wood et al*.* constructed Ir single-atom nanomaterials using electrochemical etching in CaCl_2_ solution, employing oxygen as the etching gas and neon as the imaging gas to etch polycrystalline iridium wires, demonstrating applicability in corrosive environments [59]. In simple terms, chemical etching allows for the fabrication of microstructure materials with high precision and accuracy. However, most of the etching solution is toxic and requires attention to safe handling.

As a common approach for SANs synthesis, sacrificial template method (STM) is recognized as an effective approach for creating ordered thin films using degradable templates (*e.g*., cellulose/proteins) to control film morphology and dimensions [60]. The process initiates with template selection, followed by polymer coating to stabilize the template structure. Upon achieving target thickness, mild thermal/chemical treatment induces template decomposition, yielding patterned films that direct atomic arrangement for SANs synthesis [61]. For example, Shin et al*.* presented a generalized method for the preparation of SANs on metal, metal oxide, and chalcogenide nanosheet (NS) carriers, achieving high metal loadings up to 3.94 wt% by utilizing *N*-doped graphene as a spatially confined sacrificial template [62]. While enabling microstructural precision, template-derived architectures remain constrained by template properties, limiting exact structural customization.

Beyond the aforementioned methods, there are numerous alternative “top-down” strategies for SANs synthesis, such as electrochemical stripping deposition methods [22, 63], dangling bond trapping strategies [64]. However, the current reported “top-down” strategy still exhibits inherent limitations in process maturity, with challenges in achieving precise control over particle size and morphology [65]. Moreover, this method etches large-size materials into small-size nanomaterials, and the remaining material may be wasted; it also limits the microscopic morphology of the target material and lacks atomic-scale spatial control for tailored nanostructure engineering. In practice, equal attention should be paid to ensuring the link among proper support and other possible support effects and structural changes. Existing protocols could be enhanced through systematic exploration of alternative metal precursors, modified reaction parameters, and diversified substrate selections [66].

According to the primary analysis above, the synthesis of SANs can be accomplished by reasonably tailoring “bottom-up” and “top-down” strategies. The choice of synthesizing SANs depends on factors such as available raw materials, existing facilities, potential applications, other environmental and economic constraints. To determine an appropriate synthesis method, it is of paramount importance to have a comprehensive understanding of all the underlying mechanisms of each synthesis process. The “top-down” strategy is precise but costly and suitable for basic research, while the “bottom-up” strategy is efficient and easy to scale up, which is closer to industrial applications. The future development direction may lie in combining the advantages of both, and exploring new paths such as green solvents and biocompatible precursors to balance the dispersion, stability and functionality of single atoms. As nanotechnology becomes better understood and develops rapidly, it is hoped that the challenges faced modern society will be solved.

## Applications of SANs to Environmental Pollutants

Environmental pollution is regarded as one of the most serious threats to public health and hinders the harmonious development of society. In this context, the development of efficient early-warning, traceability, and regulatory technology for environmental pollutants is of great significance and urgency. As an emerging nanotechnology, various SANs were developed and used in the monitoring and control of different environmental pollutants due to their excellent atom utilization, good stability, relatively low toxicity, etc*.* [11, 12]. The timely review and summary of the important progress on SANs-based monitoring and control of environmental pollutants is bound to effectively promote the further applications of SANs in controlling environmental pollution for safeguarding environmental health. In this section, we focus on the main progress of SANs-based monitoring and control in the environmental pollutants of phenolic & gaseous carcinogens, organic dyes, pesticide residues, medical drug residues, microbial hazards, and heavy metals.

### Applications of SANs to Phenolic & Gaseous Carcinogens

Accumulating evidence indicates that exposure to phenolic or gaseous carcinogens could induce irreversible damage to biological systems [2, 14]. To address these risks, the development of efficient monitoring platforms and remediation strategies for trace-level residues of phenolic and gaseous carcinogens in environment-related matrices has become imperative. In this subsection, the emphasis focuses on the applications of SANs in monitoring and control of carcinogenic substances related to phenolic derivatives and gaseous compounds.

**Fig. 3:**
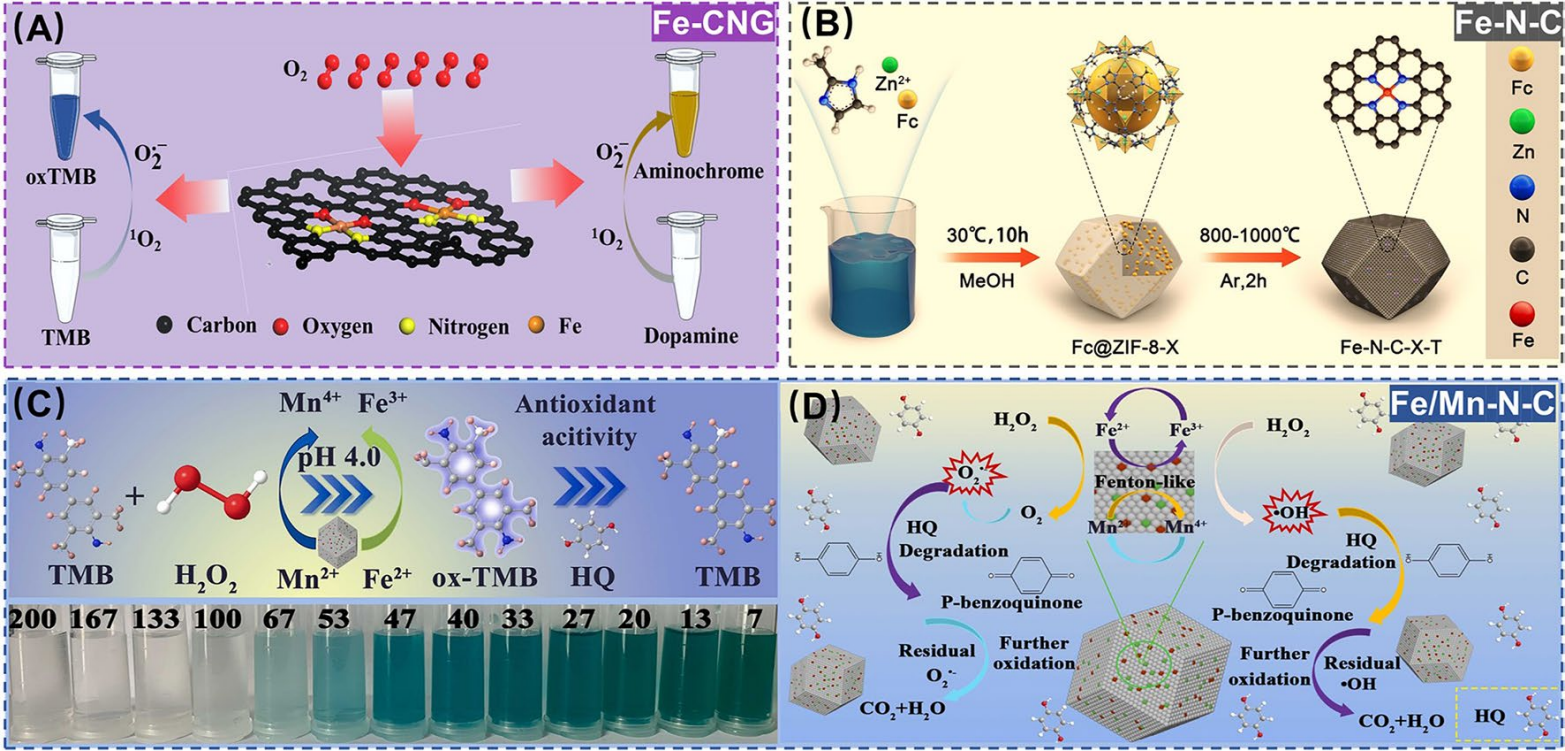
**A** Schematic diagram of the preparation of Fe-CNG for detection of HQ. Adapted with permission from Ref. [71]. Copyright 2024, Springer. **B** Schematic diagram of the preparation of Fe-N-C for degradation of BPA. Adapted with permission from Ref. [73]. Copyright 2023, Elsevier. **C, D** Schematic diagrams of the preparation of Fe/Mn-N-C for detection and degradation of HQ. Adapted with permission from Ref. [74]. Copyright 2023, Elsevier

As one of the most prevalent organic carcinogens, phenolic compounds are extensively utilized in plastics, pesticide additives, food packaging, etc. [69]. However, their pollution to environment has raised global concerns due to severe health risks, including endocrine disruption and carcinogenicity [70]. With this background, Chu et al*.* synthesized single-atom Fe-based SANs (Fe-CNG) with oxidase-like (OXD-like) activity via pyrolysis, which was next engineered to develop a colorimetric assay toward hydroquinone (HQ) residues by exploiting HQ to selectively inhibit the Fe-CNG OXD-like activity for suppressing the oxidative bluing of 3,3’,5,5’-tetramethylbenzidine (TMB) (Fig. 3A) [71]. Meanwhile, Wang et al*.* reported Fe-N_x_ single-atom composites-based SANs (FeSA-NEPBC) leveraging electron transfer-mediated oxidation to achieve the efficient degradation of 4,4-sulfobisphenol (BPS) within 40.0 min in tap water and surface water, demonstrating > 90% removal efficiency [72]. To better understand the influencing factors and mechanisms for governing phenolic pollutants by Fe-based SANs, Huang et al*.* successfully prepared a series of Fe-based SANs (Fe-N-C) through pyrolysis of iron monomers anchored on nitrogen-doped porous carbon substrates. The systematic investigation revealed that the degradation rate of Fe-N-C to bisphenol A (BPA) was correlated with the modification of the iron monomers and the higher specific surface area of the precursors. Furthermore, the formation of high-valent iron oxides (Fe(IV) = O) and interfacial electron transfer were revealed by density-functional theory (DFT) calculations, which led to a comprehensive and in-depth study of the mechanism of BPA degradation (Fig. 3B) [73]. The above SANs of Fe-N-C are mainly obtained through the nitriding Fe/C composite salts at high temperatures, and the carbon materials are prone to collapse due to the contraction and unstable bonding after pyrolysis, which tends to break the Fe-N_x_ bonds, resulting in poor stability of the Fe-N-C catalysts. For this, Liu et al*.* exploited the sacrificial template method to develop a novel three-dimensional (3D) hierarchical porous Fe/Mn-N-C-based SANs with peroxidase-like (POD-like) and superoxide dismutase-like (SOD-like) for simultaneous monitoring and control of HQ. The bimetallic Fe/Mn-N-C nanozymes possessed excellent catalytic activity and chemical stability, which paved a feasible pathway for simultaneous monitoring and control of environmental pollutants (Fig. 3C, D) [74].

**Fig. 4:**
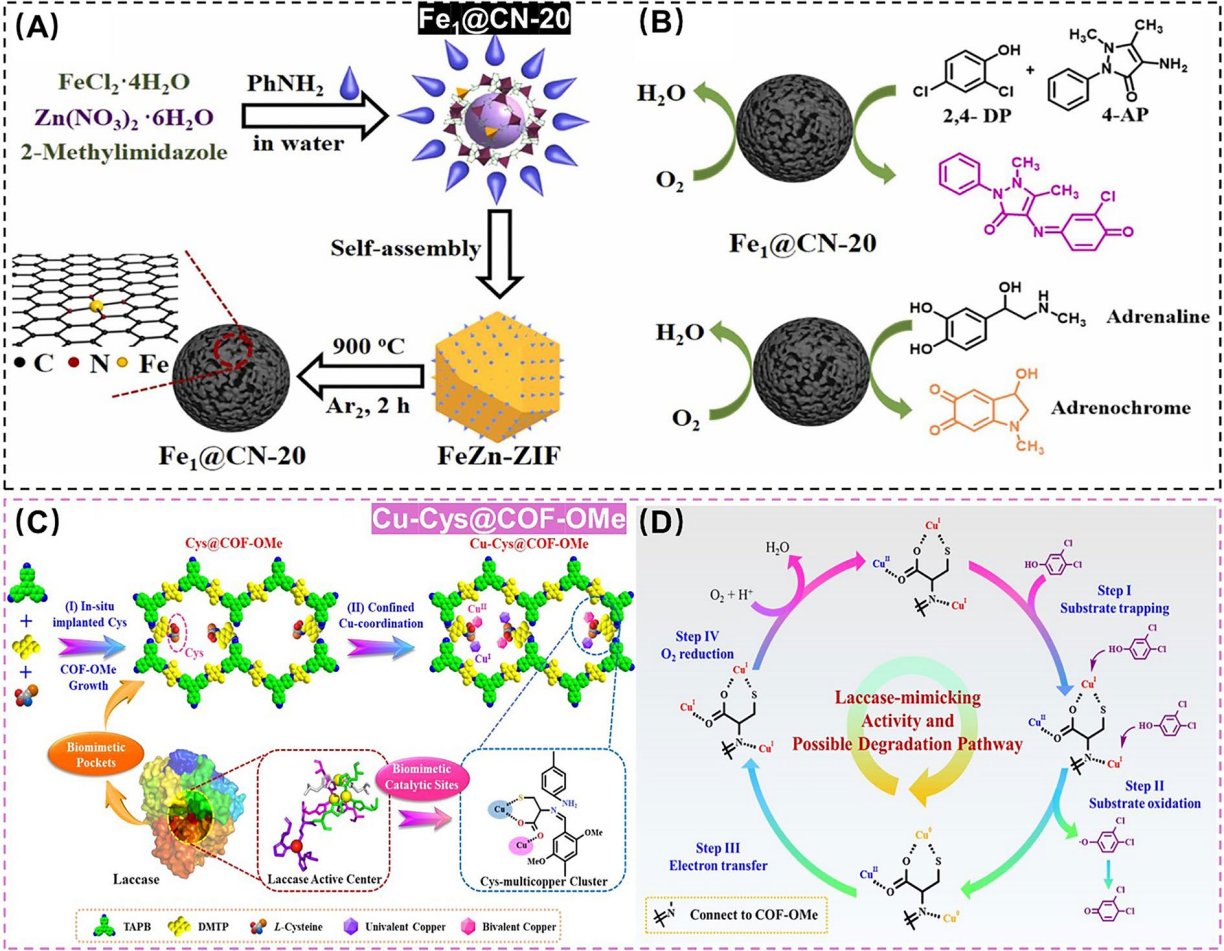
**A, B** Schematic diagrams of the preparation of Fe_1_@CN-20 for detection and degradation of phenolic compounds. Adapted with permission from Ref. [77]. Copyright 2022, Elsevier. **C, D** Schematic diagrams of the preparation of Cu-Cys@COF-OMe for degradation of phenolic compounds. Adapted with permission from Ref. [80]. Copyright 2022, Elsevier

Since a range of phenolic compounds can be oxidized to water by laccase-induced four-electron reduction process in O_2_, SANs with laccase-like activity has gained recognition as an green nanozyme to design the efficient monitoring and control strategies toward the residual phenolic pollutants as well [75, 76]. A typical example is that Lin et al*.* proposed for the first time a Fe single-atom-anchored nitrogen-doped carbon-based SANs (Fe_1_@CN-20) as a laccase mimic, which was applied to the monitoring and control of a range of phenolic compounds with a good stability even under extreme conditions (Fig. 4A, B) [77]. However, the flexible ligand coordination and intermolecular stacking enable Fe_1_@CN-20 to be low specific surface areas and non-porous structures, which is not conducive to the diffusion of reactants and the exposure of catalytic sites [78, 79]. Additionally, inspired by the unique enzyme active site and key substrate capture site of laccase, Tang et al. constructed a mesoporous biomimetic covalent organic framework (COF)-based SANs (Cu-Cys@COF-OMe), which could effectively capture and catalyze the oxidation of the residual phenolic pollutants. This synergistic biomimetic effect will significantly improve the enzymatic activity and stability of COF-based SNAs for the degradation of the residual phenolic pollutants in environment-related matrices (Fig. 4C, D) [80].

**Fig. 5:**
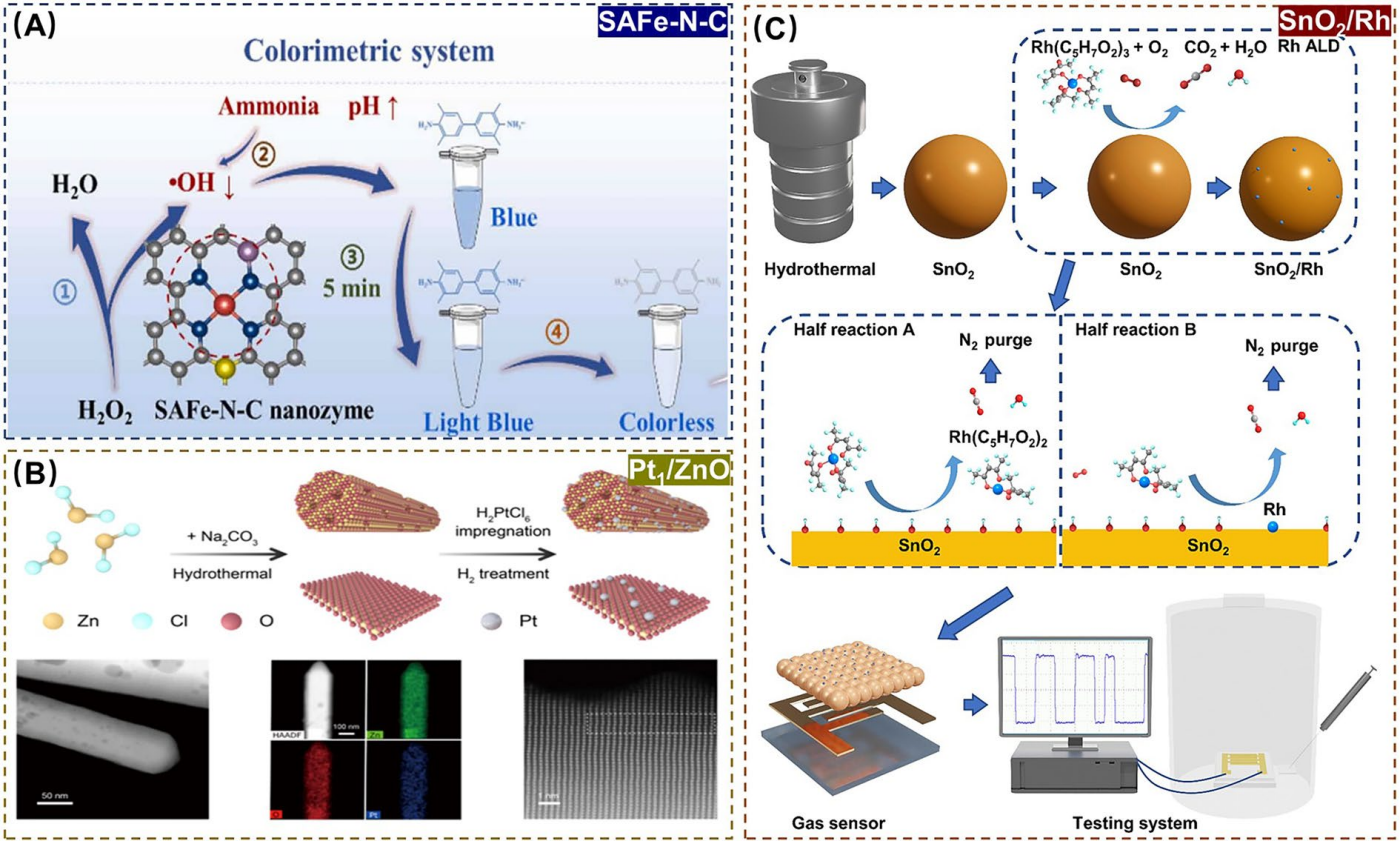
**A** Schematic diagram of the preparation of SAFe-N-C for detection of VA. Adapted with permission from Ref. [83]. Copyright 2023, Elsevier. **B** Schematic diagram of the preparation of Pt_1_/ZnO for detection of TEA. Adapted with permission from Ref. [84]. Copyright 2023, American Chemical Society. **C** Schematic diagram of the preparation of SnO_2_/Rh for detection of formaldehyde. Adapted with permission from Ref. [32]. Copyright 2023, Elsevier

In contrast to phenolic carcinogens, gaseous carcinogens present in contaminated air pose distinct health risks through prolonged respiratory exposure, such as probably inducing pulmonary tissue damage and potentially triggering cell mutation and carcinogenesis [81]. For instance, trimethylamine and dimethylamine with a putrid odor, characteristic volatile amine (VA) pollutants derived from the environmental microorganisms, belong to the precursors of the carcinogen nitrosamine and represent prototypical gaseous carcinogens [82]. Recently, the development of SANs-based methods for efficient detection of such hazardous substances has emerged as a prominent focus in environmental health research. As a typical example, Song et al*.* successfully created a colorimetric and fluorescent assay for dual-signal detection of VA using Fe-based SANs (SAFe-N-C) and carbon quantum dots (CDs). SAFe-N-C with high POD-like activity oxidized colorless 3,3’,5,5’-tetramethylbenzidine to a blue oxidation product (oxTMB). Subsequently, the fluorescence signal of CDs is burst due to the internal filtering effect (IFE) between oxTMB and CDs. However, VAs could lead to a reduction in oxTMB, which restores the fluorescence of CDs. This method has a LOD as low as 0.0838 ppm, which is lower than that of conventional gas chromatography with LOD 0.025 ppm and ambient low alarm value of 6.6 ppm (Fig. 5A) [83]. Moreover, Liu et al*.* reported a gas sensor for triethylamine (TEA) detection based on a ZnO nanorod supporting Pt-based SANs (Pt_1_/ZnO). The oxygen vacancies in Pt_1_/ZnO promoted oxygen adsorption and activation, while the formation of Pt-N bonds between TEA and Pt single atoms made TEA adsorption and activation easier, together contributing to the high sensitivity, fast response and recovery of TEA (Fig. 5B) [84]. In addition, the monitoring of volatile organic compounds (VOCs) is receiving increasing attention, in which case there is an urgent need for the development of advanced assays capable of rapid and selective adsorption detection of other gaseous carcinogens. Given this, Li et al*.* prepared Au-based SANs (Au_1_) by photochemical reduction and then modified them on In_2_O_3_ porous nanospheres (PNS) in order to obtain hybrid Au_1_/In_2_O_3_ gas sensing materials, which were successfully used for ultrafast and highly sensitive detection of CO with a LOD as low as 10.0 ppm, which is lower than the standard low-level alert value of 30.0 ppm [85]. Zhou et al*.* adopted ALD strategy to successfully prepared single-atom Rh-sensitized SnO_2_-based SANs (SnO_2_/Rh) for designing a portable formaldehyde detection device through adsorption and charge transfer between formaldehyde and SnO_2_. This assay exhibits a fast response recovery with a LOD as low as 55.0 ppb, which is comparable or even smaller than that of the conventional mass spectrometry method of 15.0–250.0 ppb, indicating a good sensitivity (Fig. 5C) [32]. The above studies have developed different SANs to enhance the performance of metal-oxide-semiconductor gas sensors, but the realization of high selectivity and response strength in chemo resistive gas sensors (CGSs) is still a major challenge. To this end, Wang et al*.* synthesized copper catalytic sites on ultrafine WO_2.72_ nanowires-based SANs (CuSA/WO_2.72_), and utilized the selective binding of toluene to Cu-based SANs (Cu SAs) to facilitate the adsorption of toluene on WO_2.72_ nanowires for achieving the highly selective detection of toluene at ultra-low concentrations [86].

The application of SANs in monitoring and control of phenolic and gaseous carcinogens has great potential. However, there is limited literature on SANs-based multi-gas adsorption and detection so far. Future research directions may primarily focus on integrating the detection functions of various gas pollutants to enable simultaneous monitoring of multiple gas components with SANs-based methods. This probably enhances the applicability and intelligence of these SANs-based systems [87]. Additionally, the emphasis may be placed on applications in SANs-based portable and wearable devices, such as SANs-based personal health monitoring and indoor air quality testing [88].

### Applications of SANs to Organic Dyes

Organic dyes are widely used coloring agent in textiles, leather, paper and other industries. Although organic dyes are very useful in providing diverse and aesthetically pleasing colors for products, they can also be released into water bodies, soil or air during production and disposal, thus posing serious hazards to the environment and humans [85, 89]. Therefore, it is necessary to monitor and control the residual organic dyes in environment-related matrices.

**Fig. 6:**
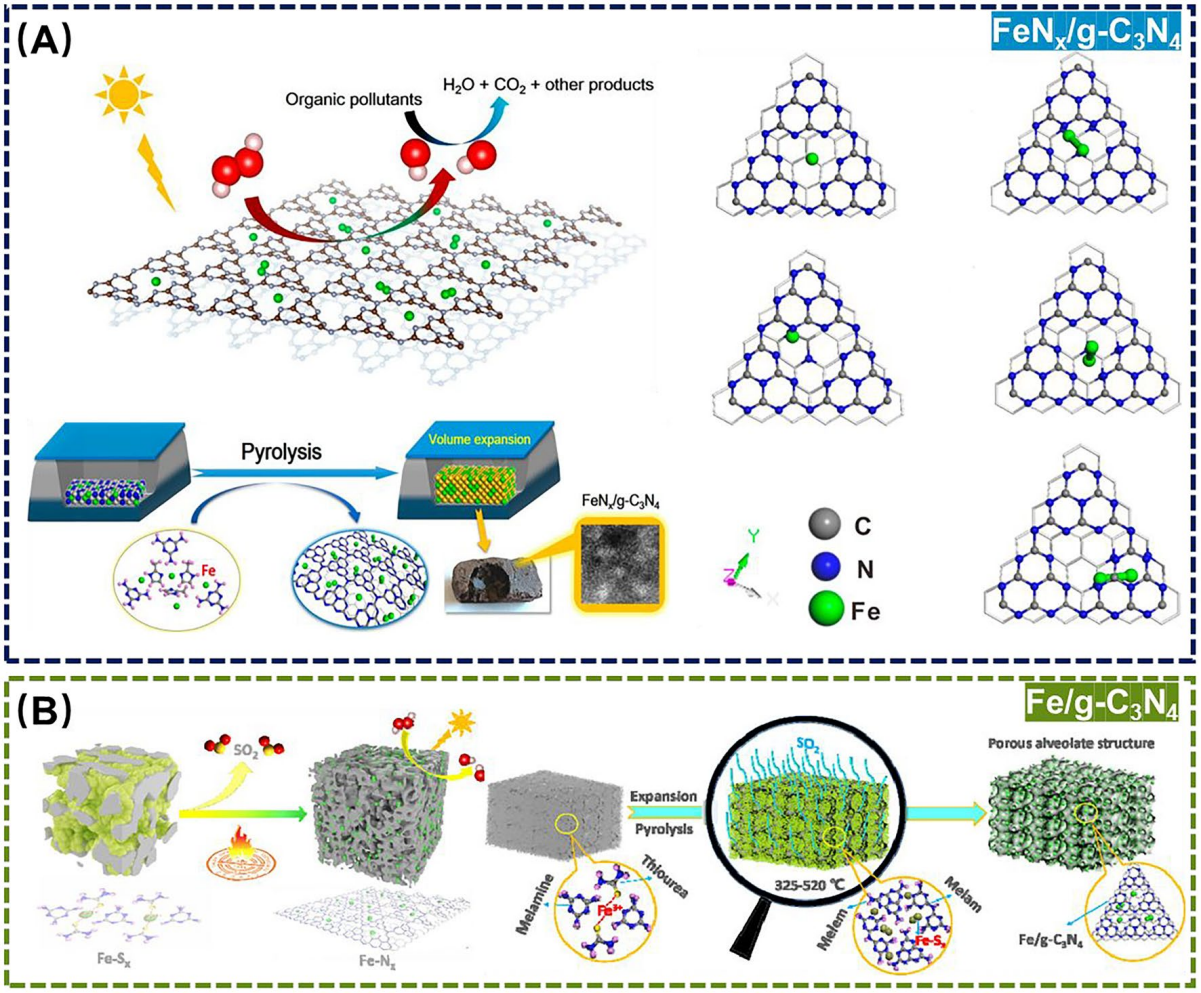
**A** Schematic diagram of the preparation of FeN_x_/g-C_3_N_4_ for degradation of MB, RhB, and MO. Adapted with permission from Ref. [94]. Copyright 2018, American Chemical Society. **B** Schematic diagram of the preparation of Fe/g-C_3_N_4_ for degradation of organic dyes. Adapted with permission from Ref. [95]. Copyright 2020, Elsevier

Photocatalytic activity originates from the transition of electrons from valence band to conduction band under the light, forming highly active electron-hole pairs, and the electron–hole pairs migrate to the surface to form reactive oxygen species (ROS) for decomposing organic and inorganic compounds [90, 91]. As we know, Fe-based SANs are widely used for the efficient degradation of organic dyes in environment-related matrices because of their environmentally friendly, efficient and non-secondary pollution properties [92]. Driven by these, combining photocatalytic substrates and Fe-based SANs is an effective way to degrade organic pollutants. As an important photocatalyst, C_3_N_4_ (especially graphite-phase carbon nitride g-C_3_N_4_) has a wide range of excellent properties to make it outstanding in photocatalysis [93]. For example, An et al*.* employed one-step pyrolysis to synthesize dense ultrasmall clusters and monatomic Fe sites embedded in g-C_3_N_4_ for obtaining Fe-based SANs, which successfully degraded methylene blue (MB), rhodamine B (RhB), and methyl orange (MO) in environmental samples under the light (Fig. 6A) [94]. However, most of the catalysts supported by g-C_3_N_4_ as the carrier are bulky in structure, small specific surface area, and low catalytic efficiency from g-C_3_N_4_-encapsulated their active sites. For this, the same group again used one-step pyrolysis to synthesize porous honeycomb g-C_3_N_4_-surpported SANs with high and increased Fe density and active sites, which exhibited excellent activity and reusability for degradation of a variety of organic dyes (*e.g.*, MB, RhB, phenol and MO) (Fig. 6B) [95]. Furthermore, Li et al*.* proposed a strong organometallic complex-carrier interaction to combine a mild heat treatment strategy for the in situ growth of synthetic Fe single atoms on the exfoliated g-C_3_N_4_, which resulted in the almost complete degradation of RhB within 30.0 min because of the large surface area, highly exposed Fe active sites, and synergistic effect between FeN_x_ and g-C_3_N_4_ carriers [96].

**Fig. 7:**
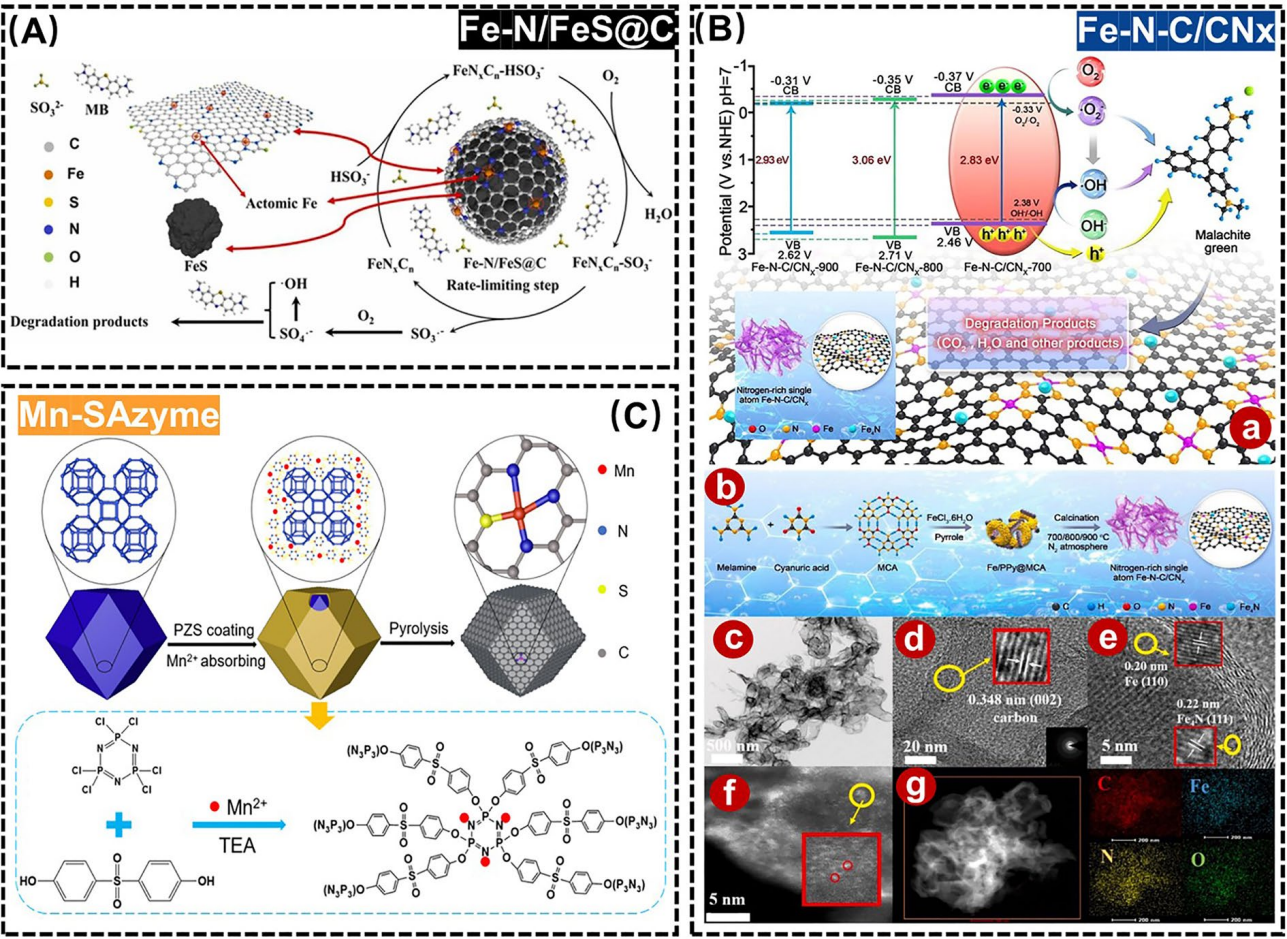
**A** Schematic diagram of Fe-N/FeS@C degradation of MB. Adapted with permission from Ref. [92]. Copyright 2021, Elsevier. **B** (**a**) Schematic diagram of Fe-N-C/CNx for degradation of MG. (**b**) Schematic illustration of preparation of the Fe-N-C/CNx hybrids by calcining the Fe/PPy@MCA composite. (**c**) Transmission electron microscopy (TEM), (**d**, **e**) High-resolution transmission electron microscopy (HR-TEM), (**f**) High-angle annular dark-field scanning transmission electron microscopy (HAADF-STEM), and (**g**) Energy-dispersive X-ray spectroscopy (EDX) images. Adapted with permission from Ref. [99]. Copyright 2022, Elsevier. **C** Schematic diagram of Mn-SAzyme for degradation of MB. Adapted with permission from Ref. [103]. Copyright 2023, American Chemical Society

Although the aforementioned Fe-based SANs work well in the degradation of organic dyes, there are still shortcomings, such as the inability of their oxides to function in acidic media and the aggregation and leaching of Fe atoms [97, 98]. To solve this problem, Wu et al*.* synthesized a novel structural composite (Fe-N/FeS@C) by pyrolysis of mechanically pretreated lignosulfonate and iron nitrate solid mixtures, which could degrade MB over a wider pH range (Fig. 7A) [92]. Meanwhile, Huang et al*.* developed a Fe–N-C-based SANs (Fe-N-C/CN_x_) embedded in a carbon nitride network, which possessed excellent photocatalytic performance because the Fe_4_N active sites and Fe-N-C monoatoms were uniformly embedded and dispersed in the CNx network, which could effectively inhibit the aggregation of metal components, and the removal efficiency of malachite green (MG) was as high as 92.1% under visible light (Fig. 7B) [99]. Despite this, there are few reports on the coexistence of SANs with densely exposed and dispersed bimetallic-N_x_ sites. In this regard, Chen et al*.* reported a novel Fe-Bi bimetallic MOF-derived carbon-loaded Fe-N_4_ and Bi-N_4_ bis-site FeBi-NC SANs, in which the introduced Bi not only acts as an active substance during the synthesis of FeBi-MOF, but also expands the distance between neighboring Fe atoms in the FeBi-MOF, prevents metal clustering during the carbonation process, and the FeBi-NC completely removes RhB within 5.0 min [100].

In addition to the above approaches, monitoring and control of organic dyes using the POD-like activity of SANs is one of the hotspots of current research. For example, Chen et al*.* successfully synthesized a novel Fe-based SANs (Fe-SAs@FNC) based on individual Fe atoms on fluorine-doped ultrathin carbon nanosheets by polymer-assisted heating, which oxidized colorless TMB to produce a blue product in the presence of hydrogen peroxide (H_2_O_2_), leading to the establishment of a new method for the colorimetric assay of *p*-phenylenediamine (PPD) in hair dyes [101]. To optimize the coordination environment, reduce the energy barrier of active intermediates, and enhance catalytic performance, Feng et al*.* pioneered the rational design and synthesis a *B*-doped Zn-N-C (ZnBNC) POD-like active SANs. This innovation enabled the development of a colorimetric sensing platform for PPD detection by inhibiting the oxidation of TMB through PPD [102]. Apart from the monitoring of organic dyes, SANs with POD-like activity can effectively degrade organic dyes as well. As a typical example, Feng et al*.* synthesized Mn-cantered,* N*, *P*and *S*-doped Mn-based SANs (Mn-SAzyme), which can be used to degrade 90% of MB in very small quantities and can accommodate a wide pH range (Fig. 7C) [103]. However, most of the reported SANs are not efficiently degraded due to the limitation that the internal active sites cannot directly contact the contaminants. To address this issue, Wu et al*.* developed a three-dimensionally ordered hierarchical micro-mesoporous-macroporous *N*-doped carbon matrix (3DOM Fe-N-C) with atomically dispersed Fe sites for efficient RhB degradation. This hierarchically ordered porous structure could accelerate the mass transfer and improve the accessibility of the active site [104].

Although SANs-based techniques have achieved good results in the monitoring and control of organic dyes, most of them are based on photocatalytic technology. It is well-known that photocatalytic technology mainly relies on a specific light source such as UV light, and for some dyes, there may be a problem of low light-source utilization efficiency. Moreover, photocatalytic reactions need to be carried out under specific reaction conditions, such as temperature and pH, which makes the operating conditions more demanding [90, 91]. To address the issues of light source utilization efficiency and catalyst activity in SANs-based photocatalytic technology, future research could focus on the development of new and efficient SANs to improve the efficiency and stability of photocatalytic technology. In addition, the comprehensive use of multiple degradation techniques, such as the combination of photocatalytic degradation technology and microbial degradation technology, to achieve complementary advantages and improve the efficiency and effectiveness of SANs-based techniques for organic dye degradation [93].

### Applications of SANs to Pesticide Residues

Pesticides used to control crop pests and diseases are frequently found in living organisms, soils and even aquatic ecosystems [105, 106], and can lead to environmental pollution, ecological impacts and health risks [7]. Given this, the development of more environmentally friendly methods and advanced materials for monitoring and degrading pesticide residues remains a great challenge.

**Fig. 8:**
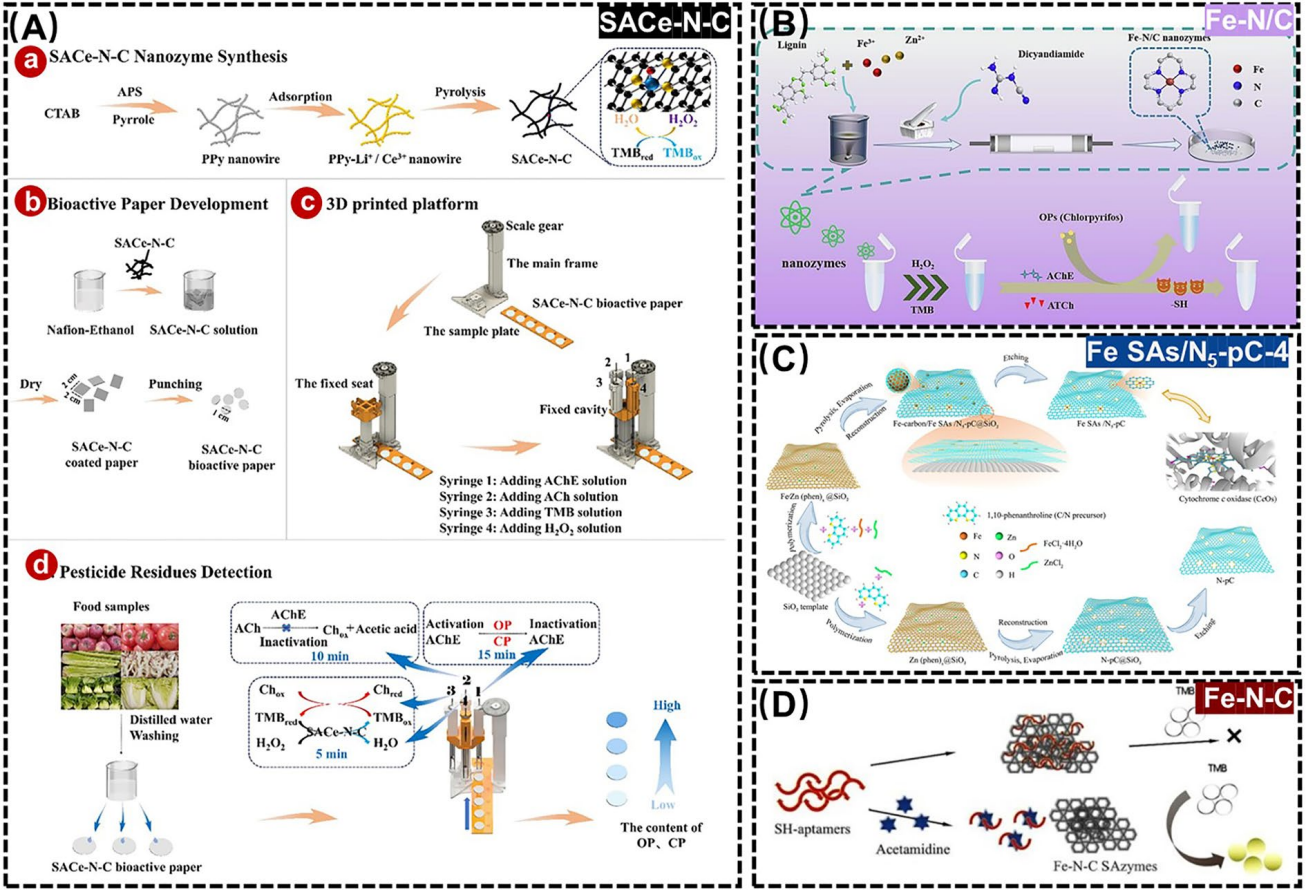
**A** Schematic diagram of SACe-N-C for detection of OPs. Adapted with permission from Ref. [108]. Copyright 2022, Elsevier. **B** Schematic diagram of Fe-N/C for detection of chlorpyrifos. Adapted with permission from Ref. [109]. Copyright 2023, Elsevier. **C** Schematic diagram of Fe SAs/N_5_-pC-4 for detection of OPs. Adapted with permission from Ref. [111]. Copyright 2022, American Chemical Society. **D** Schematic diagram of Fe-N-C for detection of Ace. Adapted with permission from Ref. [112]. Copyright 2023, Elsevier

In recent years, novel SANs possessing natural enzyme activity have been widely used for rapid detection of pesticide residues due to their high activity and good stability [107]. For example, Song et al*.* designed a Ce-based SANs (SACe-N-C) to successfully monitor organophosphorus pesticides (OPs) in the environment-related matrices (*e.g*., fruits & vegetables) within 30.0 min through a cascade catalytic reaction of its POD-like activity with acetylcholinesterase (AChE, Fig. 8A) [108]. Wang et al*.* took the one-pot pyrolysis of alkaline lignin to prepare a Fe-based SANs (Fe-N/C) with POD-like activity, which was then designed a colorimetric assay for monitoring chlorpyrifos in soil by exploiting the inhibition of chlorpyrifos to AChE activity for restoring the oxidative color of chromogenic substrate TMB (Fig. 8B) [109]. The above enzyme inhibition-based assays involve H_2_O_2_ as a co-reactant to generate ROS to oxidize the substrates, and the instability and high toxicity of H_2_O_2_ may hinder their practical applications in real environment-related matrices [110]. In this regard, Chen et al*.* prepared a hierarchical porous carbon nanoframework-based SANs (Fe SAs/N_5_-pC-4) with a Fe-N_5_ active center as a restriction point via a polymerization-pyrolysis-evaporation-etching strategy. On this basis, an OXD-like activity-driven colorimetric assay based on Fe SAs/N_5_-pC-4 for the residual OPs monitoring was proposed, thus effectively avoiding the use of the co-reactant H_2_O_2_ (Fig. 8C) [111]. Additionally, Yu et al*.* synthesized a class of OXD-like Fe-N-C SANs, modified with aptamers, for developing a Fe-based SANs-driven colorimetric assay to monitor acetamiprid (Ace) in water samples dependence on the chromogenic effect of TMB with a LOD as low as 16.9 nM (Fig. 8D) [112].

**Fig. 9:**
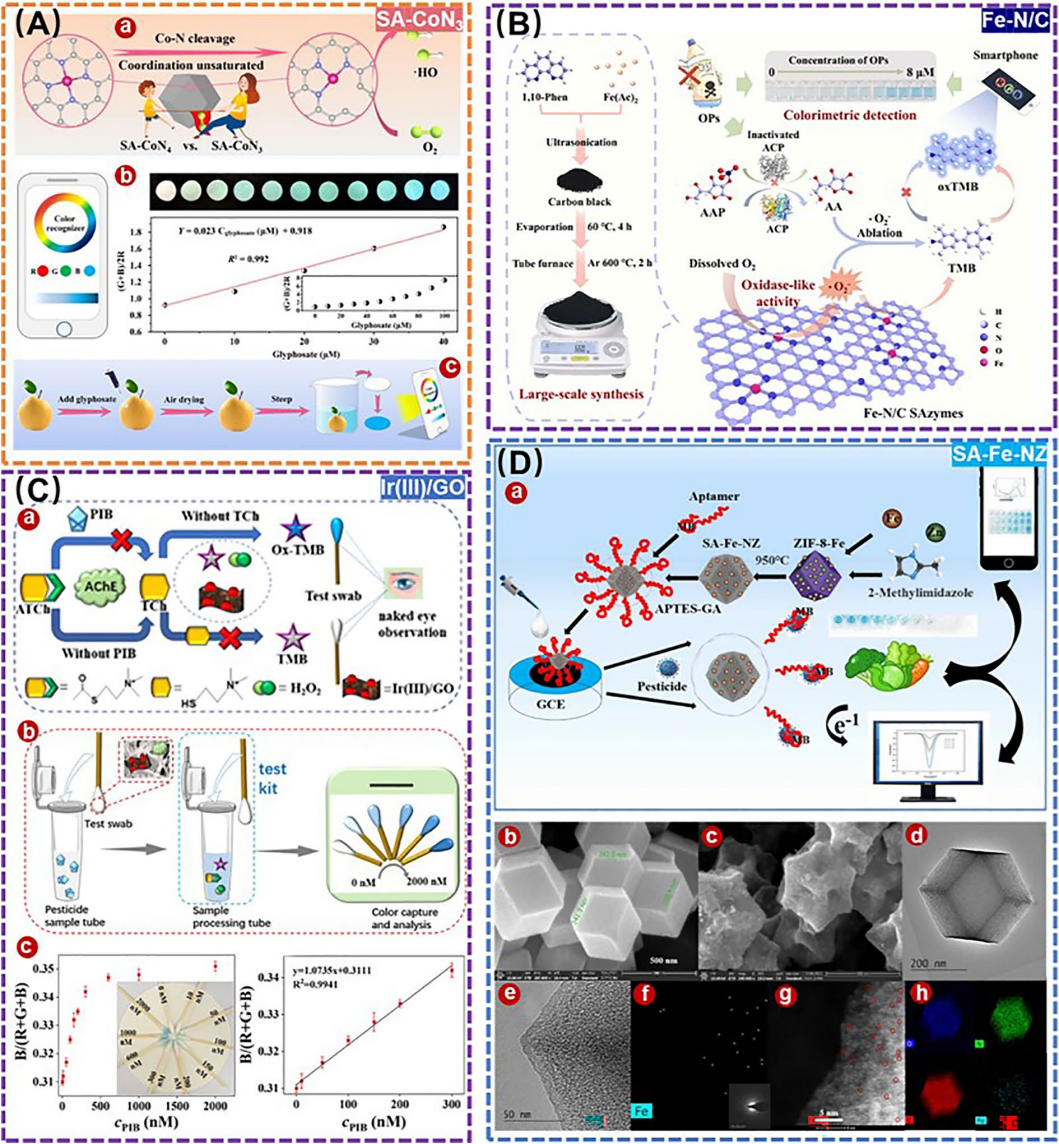
**A** Schematic diagram of SA-CoN_3_ for detection of glyphosate. **(a)** Diagram of the relationship between SA-CoN_3_ and SA-CoN_4_. **(b)** Use smartphone to detect OPs. Adapted with permission from Ref. [113]. Copyright 2023, Springer. **B** Schematic diagram of Fe-N/C SANs for detection of OPs. Adapted with permission from Ref. [114]. Copyright 2024, Elsevier. **C (a)** Schematic diagram of Ir(III)/GO for detection of perimorphs-methyl. **(b)** Exhibition of the test swabs preparation and the test steps. **(c)** Relationships between the B/(R + G + B) values and the concentrations of PIB. Adapted with permission from Ref. [115]. Copyright 2023, American Chemical Society. **D** (**a**) Schematic diagram of SA-Fe-NZ for detection of OPs. **(b)** Scanning electron microscope (SEM) image of ZIF-8-Fe; **(c)** SEM, and **(d)** TEM image of SA-Fe-NZ; **(e)** TEM image of the edge of SA-Fe-NZ; **(f)** EDX image of Fe single atoms in SA-Fe-NZ; **(g)** Aberration-corrected high-angle annular dark field-scanning transmission electron microscope (AC-HAADF-STEM) and **(h)** EDX image of SA-Fe-NZ. Adapted with permission from Ref. [118]. Copyright 2024, Elsevier

Considering the digital imaging feature enables smartphone to be a promising potable platform for onsite environmental monitoring and traceability analysis, the integration of SANs with smartphone has also attracted widespread interest in environmental applications. An example is that Liu et al. applied unsaturated cobalt nitrogen sites to embed in porous carbon for yielding a Co-based SANs (SA-CoN_3_) with an enhanced OXD-like activity. Based on this, a smartphone-assisted SA-CoN_3_-based portable colorimetric assay for onsite monitoring glyphosate residues was designed, whose work principle is resulting from the inhibition of glyphosate toward acid phosphatase (ACP) to slow down the hydrolyzation of L-ascorbic acid-2-phosphate (AAP) into ascorbic acid (AA), protecting TMB oxidation-triggered chromogenic reaction (Fig. 9A) [113]. Moreover, Zhang et al*.* achieved large-scale synthesis of Fe-N/C SANs with OXD-like activity using carbon black as a template, and designed a Fe-N/C SANs-based colorimetric assay for OPs residues. Through integration with smartphone, a visual color information-driven portable RGB analytical platform was established for onsite monitoring of OPs residues. This large-scale synthesis method for Fe–N/C SANs with excellent catalytic properties is of great importance for promoting the marketability of Fe-N/C SANs-based portable sensors toward OPs residues in real environment-related matrices (Fig. 9B) [114]. Besides, Wang et al*.* replied on the coordination reaction between Ir(III) complex and oxygen-containing groups in graphene oxide (GO) to design a Ir-based SANs ([Ir(III)/GO]) with high loading of Ir on graphene oxide (GO) nanosheets, which avoided nitrogen doping and pyrolysis procedures with a high POD-like Ir-based SANs. Encouraged by this, a color-reading APP-assisted switch-on colorimetric response-driven portable test kit for pirimiphos-methyl (PIB) monitoring was proposed by dependent on the competitive inhibition of thiocholine (TCh) (generated from the catalysis of AChE to acetylthiocholine​ (ATCh)) with the POD-like activity of Ir-based SANs, preventing TMB to be oxidized (Fig. 9C) [115]. From the above examples, it is not difficult to find that they all belong to the SANs-based single-signal analytical system toward the residual pesticides, which is susceptible to erroneous or inaccurate results [116]. In contrast, the multimodal assay offers robust immunity to interference and can provide more reliable results by cross-validating signals and a diversity of readings, improving the accuracy of SANs-driven monitoring results [117]. In view of this, Wang et al*.* took the different boiling points of Zn and Fe to dope Fe atoms on ZIF-8 to prepare SANs (SA-Fe-NZ) with POD-like activity by high temperature cracking, which was applied to establish a SA-Fe-NZ-driven colorimetric and electrochemical dual-mode detection of OPs residues in the environment-related matrices (*e.g.*, vegetables, Fig. 9D) [118].

According to the above analysis, SANs-based assays for the residual pesticides have a broad development prospect. To date, most of SANs-based analytical systems have only tested their ability to monitor the residual OPs and a few concerns focus on the monitoring of other pesticides (*e.g*., carbamate, organochlorine pesticides, etc.). Another noteworthy aspect is that some SANs can catalyze pesticides into less toxic products, thus achieving the dual role of simultaneous detection and degradation of pesticides [119], which is very valuable and has great potential for environmental pesticide management. In addition, some SANs-based assays for monitoring of pesticide residues involve the use of AChE, suffering from the drawbacks of high cost and instability to limit their more widespread use in environment-related matrices [120]. The underlying mechanism is quite complex, which covers factors such as the kinetics of inhibition of both free and immobilized enzymes, whether the inhibition is reversible or not, whether the inhibitor is competitive or not, the concentration of substrate and inhibitor, the length of incubation, and the degree of inhibition, which also lead to challenges in their environmental use. Besides, while the proof-of-concept for assays that simultaneously monitor the multiple pesticides is well established, the need to integrate multiple recognition elements within the assay remains challenging in practical environment-related applications [121].

### Applications of SANs to Medical Drug Residues

As with pesticides, medical drugs are widely found in everyday life and in the medical field, such as in animal husbandry, aquaculture, and anti-inflammatory medicine [122]. It has been reported that 30%–90% of medical drugs are released into the environment every year, and they can cause endocrine disruption and drug resistance, which are toxic to human health [123]. Therefore, it is important to develop effective remediation technologies and advanced materials to ensure effective monitoring and removal of the residual medical drugs in environment-related matrices.

**Fig. 10:**
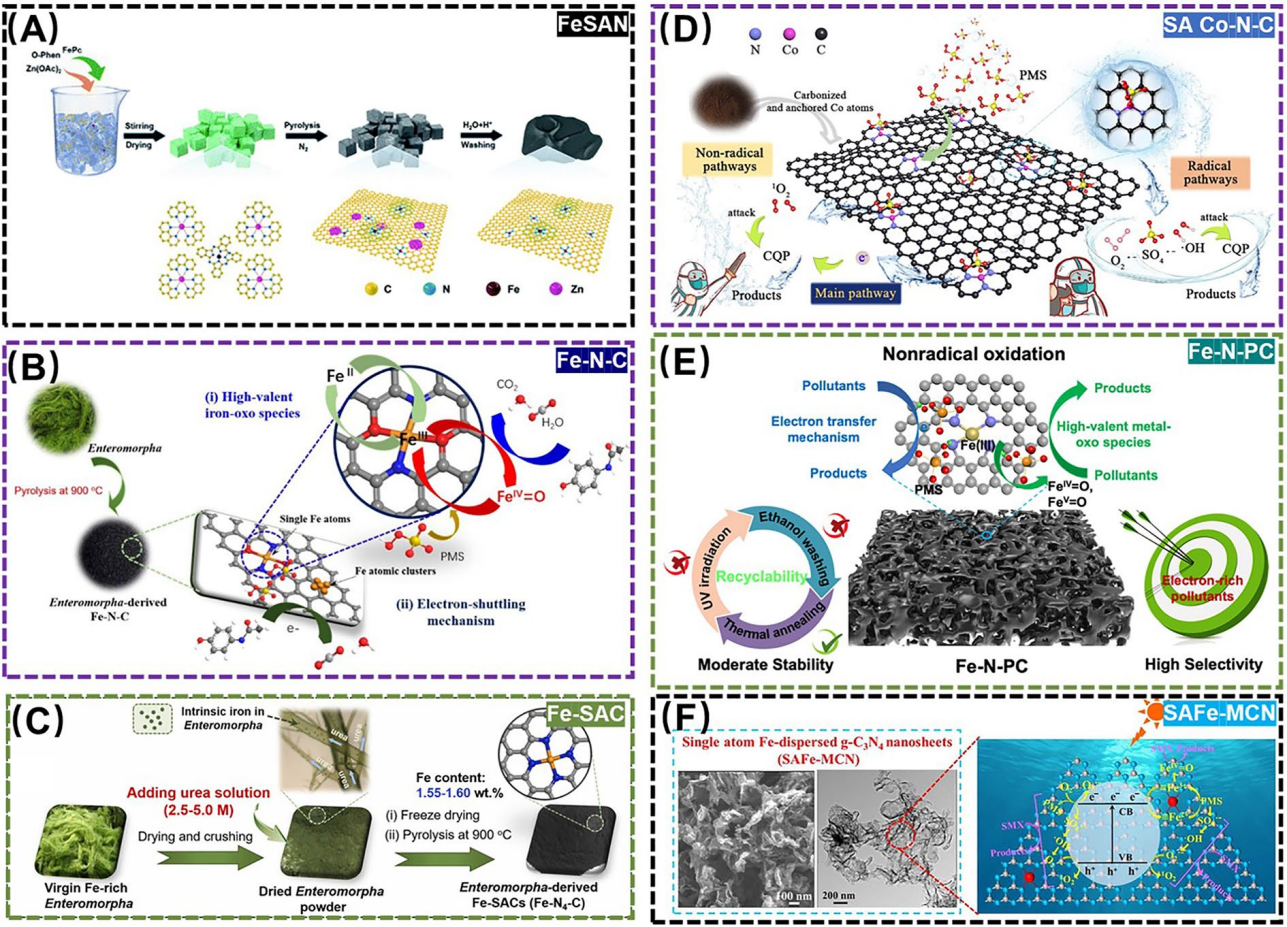
**A** Schematic diagram of FeSAN for detection of 4-AMP. Adapted with permission from Ref. [124]. Copyright 2022, Royal Society of Chemistry. **B** Schematic diagram of Fe-N-C for degradation of 4-AMP. Adapted with permission from Ref. [126]. Copyright 2021, Elsevier. **C** Schematic diagram of Fe-SAC for degradation of NPX. Adapted with permission from Ref. [127]. Copyright 2023, Elsevier. **D** Schematic diagram of SA Co-N-C for degradation of CQP. Adapted with permission from Ref. [128]. Copyright 2022, Elsevier. **E** Schematic diagram of Fe-N-PC for degradation of IBP. Adapted with permission from Ref. [130]. Copyright 2022, Elsevier. **F** Schematic diagram of SAFe-MCN for degradation of SMX. Adapted with permission from Ref. [131]. Copyright 2022, Elsevier

Such as monitoring pesticide residues, SANs also show a promising potential in monitoring and control of medical drug residues, which may be harmful to environmental health. For example, Wu et al*.* synthesized a Fe-based SANs (FeSAN) with high OXD-like activity to engineer a colorimetric monitoring of 4-acetaminophen (AMP) by the inhibitory effect of the analgesic and antipyretic drug AMP on the OXD-like activity of FeSAN (Fig. 10A) [124]. The advanced oxidation process (AOP) based on sulfate radicals is considered to be an ideal technique for degradation of various environmental pollutants by reactive species [125]. For this point, Peng et al*.* synthesized another Fe-based SANs (Fe-N-C) using seaweed and Fe/N-rich precursors and established a peroxymonosulfate​ (PMS)-activated system for degradation of acetaminophen (Fig. 10B) [126]. Moreover, Yin et al*.* utilized seaweed powder as a Fe and N-rich green raw material to synthesize a Fe-based SANs (Fe-SAC) with highly dispersed Fe monoatoms, which can completely degrade naproxen in aqueous solution within 10.0 min (Fig. 10C) [127]. Peng et al*.* prepared a biochar-derived Co-based SANs (SA Co-N-C) to develop a PMS activation system for efficiently degrading chloroquine phosphate over a wide pH range (Fig. 10D) [128]. It has been reported that some bursting agents such as L-histidine and *p*-benzoquinone can react directly with PMS in AOP process, leading to peroxide depletion and inhibiting oxidation, thereby ultimately misrepresenting the mechanism to make the results inaccurate [129]. As a case study for SANs-based strategy, Wang et al*.* developed a graded porous iron/nitrogen-doped carbons-based SANs (Fe-N-PC) for the PMS oxidation of ibuprofen, with an almost complete degradation efficiency within 30.0 min. This work aims to deepen the understanding of non-radical mechanisms and structure-oriented PMS activation in engineered carbonaceous materials (Fig. 10E) [130]. To understand whether it is the monoatomic Fe or the photogenerated electrons (e^−^)/holes (h^+^) that play a key role in persulfate activation, Zhao et al*.* synthesized a Fe-dispersed g-C_3_N_4_-based SANs with a Fe-N_4_ coordination structure for photocatalytic activation of PMS to degrade sulfamethoxazole (SMX). This work revealed the dominant role of Fe single atoms in PMS activation and SMX removal, and elucidated the contribution of photogenerated electrons (e^−^) and holes (h^+^) to the enhanced catalytic performance of the SANs in PMS activation (Fig. 10F) [131].

**Fig. 11:**
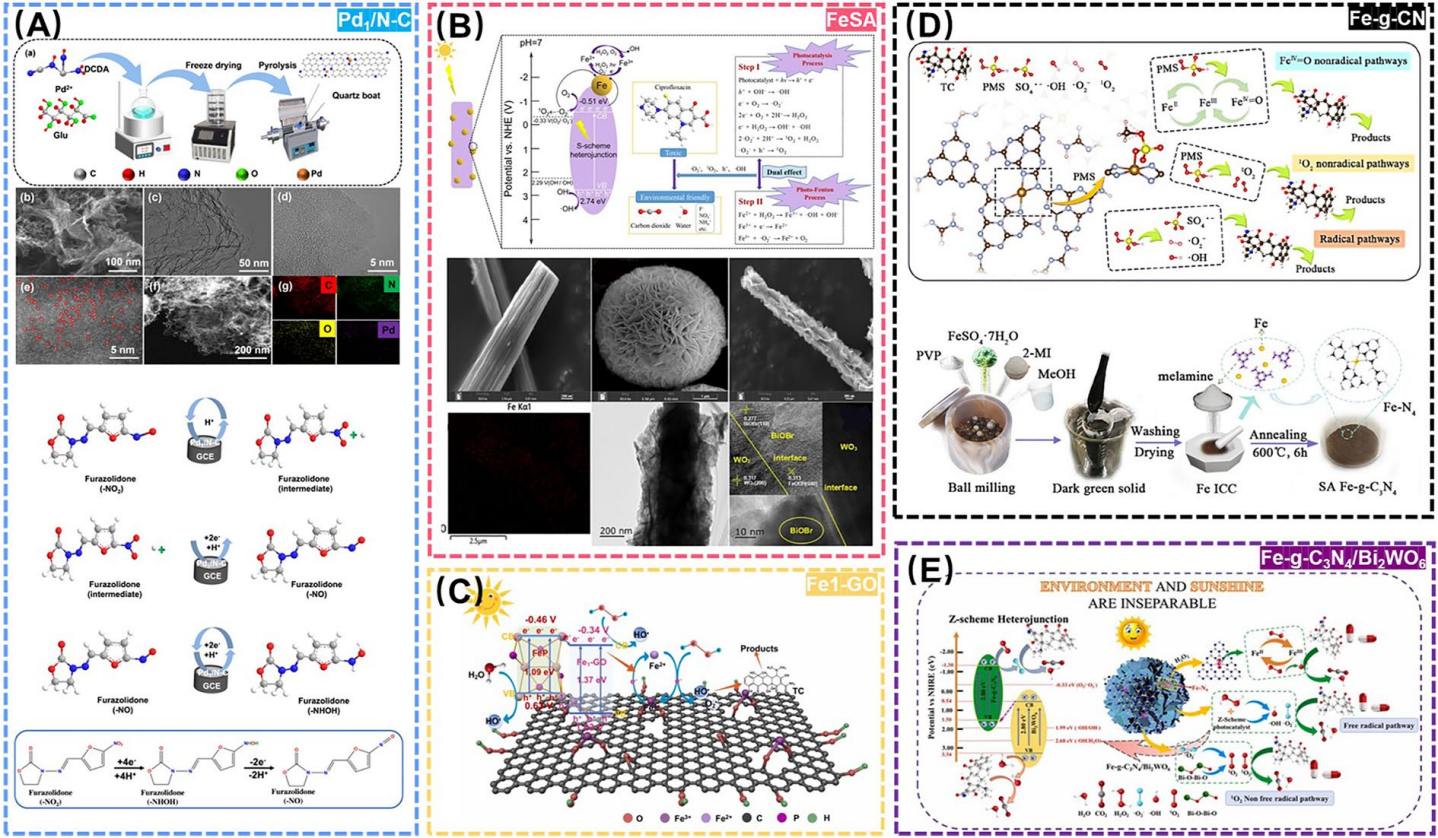
**A** Schematic diagram of Pd_1_/N-C for detection of FZD. Adapted with permission from Ref. [134]. Copyright 2023, Elsevier. **B** Schematic diagram of FeSA for degradation of CIP. Adapted with permission from Ref. [136]. Copyright 2023, Elsevier. **C** Schematic diagram of Fe_1_-GO for degradation of TC. Adapted with permission from Ref. [138]. Copyright 2023, Elsevier. **D** Schematic diagram of Fe-g-CN for degradation of TC. Adapted with permission from Ref. [140]. Copyright 2022, Elsevier. **E** Schematic diagram of Fe-g-C_3_N_4_/Bi_2_WO_6_ for degradation of degradation of TC. Adapted with permission from Ref. [141]. Copyright 2022, Elsevier

The PMS-based Fenton-like reaction as an AOP has recently shown great potential in degrading antibiotics in environment-related matrices as well. As a representative example, Chen et al*.* anchored monatomic Cu sites onto reduced graphene oxide (rGO) to acquire Cu-based SANs (SA-Cu/rGO), which could effectively degrade various antibiotics, including SMX, meropenem, and sulfafurazole via non-homogeneous activation of PMS. This study unveiled the distinct PMS activation mechanisms by rGO and SA-Cu/rGO, while demonstrating a synergistic coupling effect between the rGO carrier and the monatomic copper active sites [132]. In spite of this, problems such as narrow pH value range, sludge formation and recycling also restrict the development of this field. To break the deadlock, Yang et al*.* developed a monoatomic Fe-based SANs (Fe-ISAs@CN) immobilized by nitrogen-doped porous carbon material derived from metal-organic framework (MOF) precursors, successfully engineered for efficient degradation of sulfadiazine (SDZ) in a wide pH catalytic range and five reaction cycles [133]. Furthermore, Han et al*.* designed a Pd-based SANs (Pd_1_/N-C) by varying the metal doping-immobilized nitrogen-doped porous carbon material, which was successfully applied to the electrochemical monitoring of the residual furazolidone (FZD), manifesting a promising potential for environmental real-time monitoring and traceability of the residual antibiotics (Fig. 11A) [134].

While the above SANs-based oxidation processes have demonstrated the efficacy in monitoring and control of medical drug residues, they still suffer from several limitation including excessive reagent consumption and challenges in catalyst separation/recovery [135]. In this regard, Yu et al*.* constructed a novel photo-Fenton coupling system to efficiently degrade ciprofloxacin (CIP) in wastewater by employing a highly active Fe-based SANs as a bridge connecting photocatalysis and Fenton oxidation (Fig. 11B) [136]. However, the low catalytic activity and poor stability of this mono-metal iron composite SANs limit its practical application in complicated environment-related matrices [137]. In contrast, the special potential structure and band gap of iron phosphide (FeP) may be capable of overcoming these deficiencies. For instance, Li et al*.* constructed a novel Fe-based SANs compositing FeP and Fe single-atom graphene oxide (Fe_1_-GO), endowing a dual photo functional sites to achieve the efficient photodegradation of tetracycline (TC) with an excellent stability even after 100 cycles (Fig. 11C) [138].

**Fig. 12:**
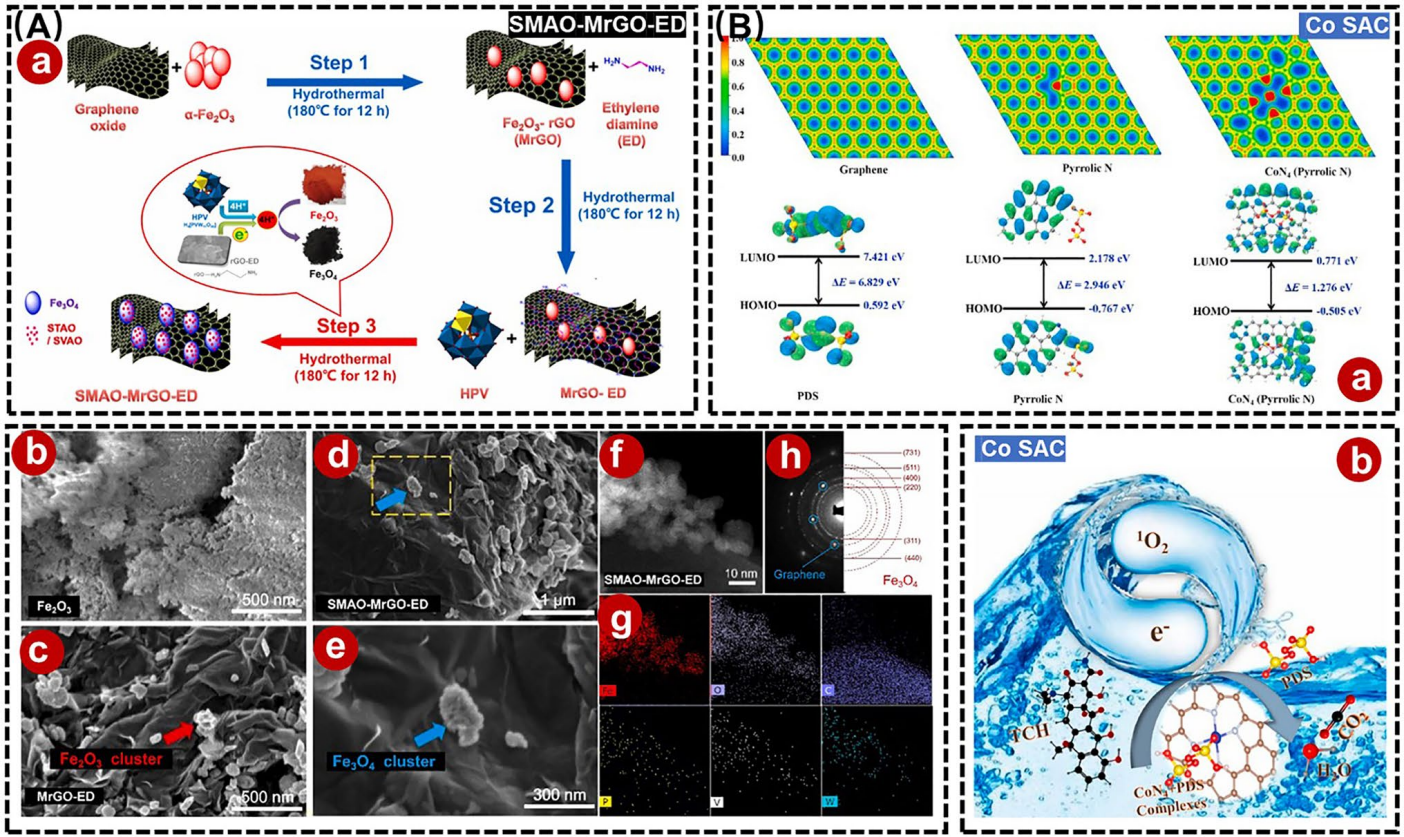
**A (a)** Schematic diagram of SMAO-MrGO-ED for degradation of CIP and IBP. Field emission scanning electron microscopy (FE-SEM) images of **(b)** Fe_2_O_3_, **(c)** MrGO-ED, **(d)** and **(e)** SMAO-MrGO-ED nanocomposites. **(f)** STEM image of SMAO-MrGO-ED and **(g)** the corresponding EDX mapping of the Fe, O, C, P, V, W elements in SMAO-MrGO-ED. **(h)** Selected area electron diffraction (SAED) pattern of SMAO-MrGO-ED. Adapted with permission from Ref. [145]. Copyright 2022, Elsevier. **B (a)** Two-dimensional charge distribution maps on different graphene models. **(b)** Schematic diagram of Co SAC for degradation of TC. Adapted with permission from Ref. [147]. Copyright 2023, Elsevier

Like some of the scenes above, g-C_3_N_4_ has been used as an anchoring carrier for metal sites to perform SANs-driven photo-Fenton processes, owing to its graphite-like structure, high thermal, chemical stability, etc. [139]. For example, Peng et al*.* anchored Fe-based SANs and Fe clusters to the g-C_3_N_4_ skeleton, which activated PMS to generate high-valent iron oxides and then efficiently degrade TC (Fig. 11D) [140]. Liu et al*.* constructed a Fe-g-C_3_N_4_/Bi_2_WO_6_ Z-type heterostructure-based SANs to degrade TC residues via a photo-Fenton process, with enhanced electron transfer and catalytic performance toward H_2_O_2_ (Fig. 11E) [141]. However, the poor electronic properties and low adsorption of g-C_3_N_4_ may also hinder the in situ decomposition of H_2_O_2_ [142]. For this, Fan et al. synthesized a Pt single-atom modification-based carbon nitride nanorod (SA-Pt/g-C_3_N_4_-K), in which the enhanced crystallinity of g-C_3_N_4_ effectively reduced the charge transfer blocking sites and increased the charge mobility, thereby demonstrating excellent POD-like activity, which successfully enabled the detection of oxytetracycline (OTC) [143]. Although the conductive support materials like g-C_3_N_4_ have shown potential in enhancing SANs performance through increasing active site density and minimizing metal size to atomic scales, the precise engineering of high-density single metal atom oxides (SMAOs) remains a formidable challenge due to their inherent instability [144]. In this case, Selvakumar et al*.* prepared a SMAO-MrGO-ED nanocomposite that provided abundant active sites for the photodegradation of ciprofloxacin (CF) and ibuprofen (IBF) with 98.43% and 98.12% activity, respectively (Fig. 12A) [145]. Yang et al*.* significantly improved the atomic utilization efficiency of SANs by implanting monatomic cobalt into polymerized carbon nitride (Co-pCN) via a bidentate ligand for effective photocatalytic degradation of OTC [146]. Meanwhile, to develop a SANs that can work effectively in cold regions or in winter, Zhang et al*.* synthesized a Co-based SANs (Co SAC) for the effective degradation of TC at 4–35 °C using straw biochar as a raw material, and this work greatly extends the application of green catalysts under cold and harsh conditions (Fig. 12B) [147].

From the above examples, the SANs-based techniques for the analysis and control of medical drug residues monitoring exhibit a promising potential in environmental monitoring and governance. However, most endeavors have only focused on monitoring the commonly antibiotic classes (*e.g.*, sulfonamides, beta-lactams, and tetracyclines). Analysis of other residual medical drugs (*e.g.,* colchicine, trimethoprim, etc*.*) is still limited [148, 149]. Moreover, most of the current reports are only in the proof-of-concept stage, while the real environment-related matrices are very complex, and the subsequent efforts in exploring SANs-based techniques to monitor and control the residual medical drugs in real environment scene should be strengthened [150]. Besides, more innovative and smart SANs should be developed for the simultaneous monitoring and degradation of the residual medical drugs in real environment scene at the same time, together with some additional environmental safety evaluation functions such as imaging, diagnostics, and governance monitoring [151].

### Applications of SANs to Microbial Hazards

Over the past decades, the pollution of microbial hazards (*e.g.*, harmful microbes, including bacteria, fungi, viruses, and their toxic by-products like mycotoxins) has become one of the leading causes to threat public health, resulting in about 7 million deaths worldwide each year [152, 153]. In particular, with the misuse of antibiotics, the emergence of antibiotic-resistant bacteria in the environment enables this threat to be more serious [154]. In this case, it is of great significance to explore the efficient strategy for monitoring and control of microbial hazards in safeguarding environmental health.

**Fig. 13:**
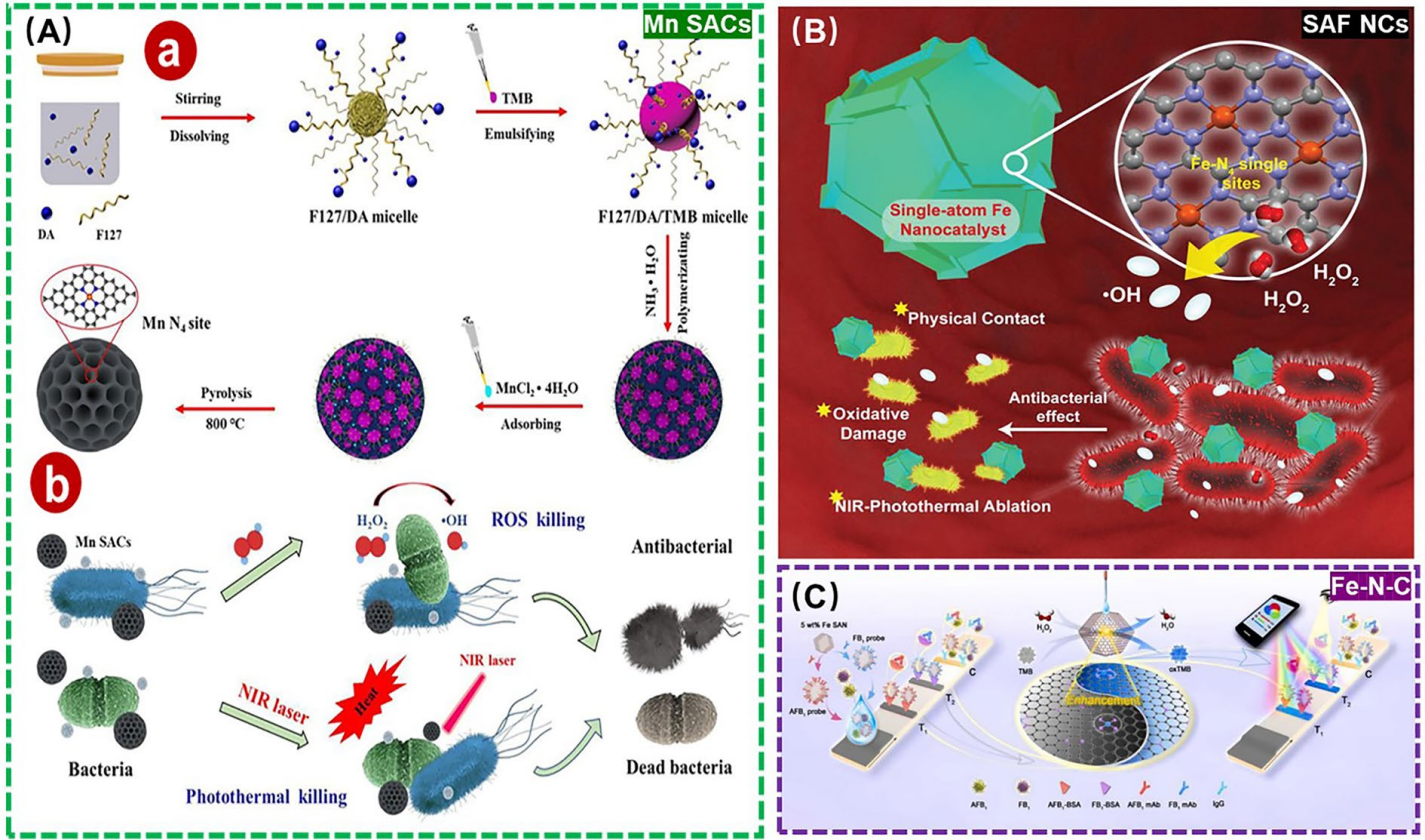
**A** (**a, b**) Schematic diagram of Mn SACs for disinfection. Adapted with permission from Ref. [160]. Copyright 2022, Elsevier. **B** Schematic diagram of SAF NCs for disinfection. Adapted with permission from Ref. [161]. Copyright 2019, Wiley–VCH Verlag. **C** Schematic diagram of Fe-N-C for detection of AFB_1_ and FB_1_. Adapted with permission from Ref. [167]. Copyright 2023, Elsevier

Recently, various enzyme-like SANs are considered to be effective antibiotics-like that utilize ROS to cause oxidative damage to the cell membranes and cell walls of harmful microbes, resulting in effective disinfection action [155]. As a prime example, Dai et al*.* adopted coordination structure optimization to develop an atomic-thickness C_3_N_4_ nanodot-supported Cu-based SANs (Cu-CNND) with a full exposure of Cu-N_3_ active site, improving the generation of hydroxyl radicals (•OH) from H_2_O_2_ and exhibiting an excellent disinfection effect of more than 99% [156]. Nevertheless, the introduced high dose of H_2_O_2_ may cause new hazards, because of its high toxicity as well [157, 158]. To address this problem, Feng et al*.* designed a spherical mesoporous Fe-based SANs (Fe-N-C SAzyme) to yield an efficient disinfection with acceptable H_2_O_2_ dose via a photothermal treatment-enhanced Fenton-like catalytic process. Notably, the high catalytic performance and high photothermal conversion efficiency of this proposed Fe-N-C SAzyme are ascribed to the large pore size, high specific surface area and carbon framework [159]. Similarly, Xu et al*.* exploited nanoemulsion assembly method to develop a spherical mesoporous Mn-based SANs (Mn SACs) based on Pluronic F127 and dopamine hydrochloride, with an enhanced catalytic activity with the help of its photothermal properties for achieving an excellent disinfection effect and biocompatibility (Fig. 13A) [160]. In addition, Huo et al*.* synthesized a nitrogen-doped carbon-supported Fe-based SANs (SAF NCs) to catalyze H_2_O_2_ for producing abundant •OH, effectively destroying bacterial cells in combination with 808 nm near-infrared laser irradiation as well (Fig. 13B) [161]. Judging from these positive research developments, SANs may provide a new opportunity to block the spread of resistant bacteria and even antibiotic resistance genes (ARGs) in the environment, safeguarding ecosystem and human health in the near future.

Except for the harmful microbes themselves, some of their toxic by-products (*e.g.*, mycotoxins) could be considered as a kind of potential environmental pollutants and pose a serious threat to the environment health as well [162–165]. In this context, Guo et al*.* developed a direct competitive immunosorbent assay based on POD-like Fe-based SANs (Fe-N-C SANs) for aflatoxin B_1_ (AFB_1_) monitoring by dependent on the colorimetric signals of TMB, with a LOD as low as 3.3 pg mL^−1^. This is the first SANs-based enzyme-linked immunosorbent assay (ELISA) for the determination of AFB_1_ [166]. Likewise, Cai et al*.* utilized pyrolysis to ferric ions-doped zeolite imidazolium skeleton-8 (ZIF-8) to synthesize POD-like Fe-based SANs (Fe-N-C SAzyme), which was employed as the chromogenic marker and catalyst in smartphone-supported lateral flow immunoassays for portable monitoring AFB_1_ and fumonisin B1 (FB_1_) with LOD of 2.8 and 13.9 pg mL^−1^, respectively (Fig. 13C) [167]. Above all, the detection and study of the harmfully microbial metabolic toxins by SANs can help to deeply understand the ecological characteristics of microbes and the production mechanisms of toxic by-products, so as to assess the environmental pollution degree, perform the traceability analysis, formulate effective environmental protection measures, and maintain the ecological balance.

Although the aforementioned examples demonstrate the promising application prospects of SANs in detection and disinfection control of microbial hazards, there are still several limitations that require improvement. For instance: (1) Current research primarily focuses on the targeting mechanisms of SANs toward microbes through electrostatic forces, pH, and surface roughness. However, the complex environmental media components can weaken these weak interaction effects, affecting the disinfection efficacy of SANs-based approaches, so more precise targeting SANs-based systems need to be developed [168, 169]. (2) While SANs have advanced rapidly in environmental analysis and disinfection, most studies focus on either detection or disinfection as separate processes, with limited comprehensive reports. There is an urgent need to explore integrated strategies that combine real-time detection with simultaneous disinfection. Such SANs-based approaches would not only prevent environmental contamination from microbial hazards but also align with the concept of environmentally sustainable development.

### Applications of SANs to Heavy Metals

Heavy metals, characterized by high density and atomic weight, are a kind of representative environmental pollutants, such as lead, cadmium, and mercury [170]. The primary environmental sources of heavy metals encompass industrial emissions, pesticides, chemical fertilizers, wastewater, etc. [171]. They usually pose severe threats to human health, because they can accumulate in the body to cause chronic poisoning, impairing organ functions, and even leading to cancer [172]. Strengthening the efficient pollution monitoring and control of residual heavy metals in environment-related matrices has been a research hotspot for a long time. In this section, the progress of SANs on this field is reviewed.

**Fig. 14:**
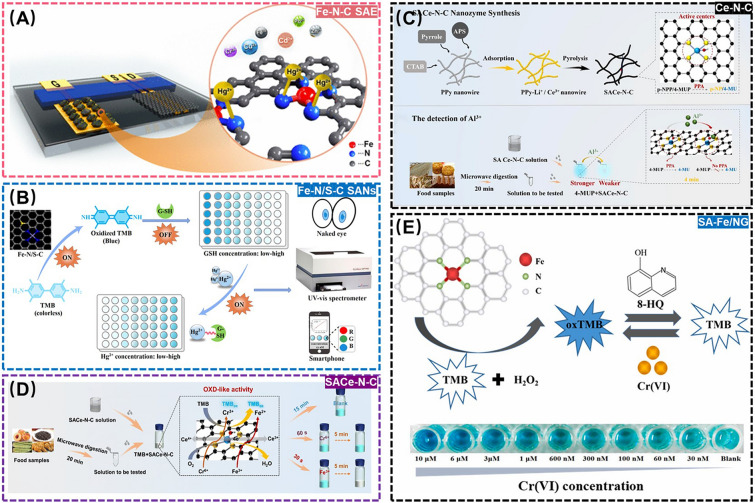
**A** Schematic diagram of Fe-N-C SAE for detection of Hg^2+^. Adapted with permission from Ref. [175]. Copyright 2020, American Chemical Society. **B** Schematic diagram of Fe-N/S-C SANs for detection of Hg^2+^. Adapted with permission from Ref. [176]. Copyright 2022, Elsevier. **C** Schematic diagram of SACe-N-C for detection of Al^3+^. Adapted with permission from Ref. [177]. Copyright 2022, Elsevier. **D** Schematic diagram of Ce-N-C for detection of Fe^3+^ and Cr^6+^. Adapted with permission from Ref. [178]. Copyright 2022, Royal Society of Chemistry. **E** Schematic diagram of SA-Fe/NG for detection and degradation of Cr^4+^. Adapted with permission from Ref. [179]. Copyright 2021, Elsevier

Mercury ion (Hg^2+^), one of the most common heavy metals in nature, is highly toxic, which can be easily converted into methylmercury to accumulate in the human body, resulting in various disorders and irreversible damages to systems such as the endocrine system, liver, kidneys, brain, and nervous system [173, 174]. In view of this point, the potential of SANs to monitor the residual Hg^2+^ in environment-related matrices has been intensively investigated. For example, Yao et al. developed a Fe-based SANs (Fe-N-C SAE) by modifying dodecahedral *N*-doped carbon to incorporate single Fe sites, to design a specific assay toward the residual Hg^2+^ through the chelation between Hg^2+^ and *N* atoms on Fe-N-C SAE, allowing a low LOD and rapid detection time of 1.0 nM and 2.0 s (Fig. 14A) [175]. In another example, Li et al. used peanut shells as the sources of C, N, and S to synthesize a nitrogen and sulfur coordinated Fe-based SANs (Fe-N/S-C SANs) with high OXD-like activity. In this design, Fe-N/S-C SANs could oxidize TMB to blue oxTMB, which was then inhibited by glutathione (GSH) to prevent the release of blue color. However, the high affinity of Hg^2+^ to GSH for forming Hg^2+^-SH complex competitively restored the oxidization of TMB into blue oxTMB. On this basis, a Fe-N/S-C SANs-driven colorimetric assay for Hg^2+^ residues monitoring was successfully developed with a LOD as low as 0.17 nM (Fig. 14B) [176].

In a related investigation, Song et al*.* engineered an exceptional phosphatase-like (PPA) cerium-nitrogen-carbon (Ce-N-C)-based SANs (SA-Ce-N-C) to catalyze inorganic phosphate for carrying out dephosphorylation. By leveraging the specific Al^3+^-induced formation of Al-O bonds with O atoms in SA-Ce-N-C structure, the PPA activity of SA-Ce-N-C was effectively inhibited. Based on this principle, a rapid SA-Ce-N-C-based fluorescence assay for Al^3+^ residues was proposed with an LOD of 22.89 ng mL^−1^. However, this LOD value is greater than the limit value of 20 ng mL^−1^ for Al^3+^ in drinking water in China, which needs further improvement (Fig. 14C) [177]. After that, the same team further explored the simultaneous detection of Fe^3+^ and Cr^6+^ residues by another Ce-based SANs (Ce-N-C SANzyme). In the presence of TMB, Fe^3+^ and Cr^6+^ significantly increased the electron conversion rates of Ce^3+^ and Ce^4+^ in Ce-N-C SANzyme, which in turn affected its OXD-like activity. Based on this, a time-resolved analytical method was constructed to monitor the residual Fe^3+^ and Cr^6+^ (Fig. 14D) [178]. The aforementioned studies concerning SANs solely enable the detection of environmental heavy metals but cannot achieve their effective elimination. Therefore, Mao et al*.* anchored Fe atoms on a single layer of 2D nitrogen-doped graphene to prepare a POD-like Fe-based SANs (SA-Fe/NG) for integration of the monitoring and removal feature toward Cr^4+^. Briefly, 8-hydroxyquinoline (8-HQ) was used as an inhibitor to prevent TMB oxidation caused by SA-Fe/NG, suppressing blue color to be produced. Attributed to the exceptional chelating capacity of 8-HQ with Cr^4+^, Cr^4+^ was preferentially removed, resulting in a return of SA-Fe/NG to oxidize TMB for releasing blue color of oxTMB. Therefore, an integrated SANs-based platform for colorimetric monitoring and removal of Cr^4+^ was developed (Fig. 14E) [179].

Based on these advancements above, SANs may offer new possibilities for efficient monitoring and controlling the residual heavy metals in environment-related matrices. Nevertheless, the following challenges still remain: (1) At present, the applications of SANs mainly focus on monitoring heavy metal ions, and few studies have applied SANs for monitoring the residual heavy metals in organic form (*e.g.*, CH_3_Hg). Meanwhile, the efforts on SANs-based removal techniques for heavy metals is not enough as well. (2) Most the current studies have focused on SANs-involved some composite nanomaterials, which aggravates the complexity of synthesis. (3) The reported SANs-based strategies for monitoring heavy metals is mainly limited to colorimetry and is susceptible to interference from other colored components in real environment-related matrices to be tested [180]. Future research directions should focus on developing more efficient and reliable SANs, optimizing their design and synthesis strategies, improving their adaptability and stability in complex real environments, and further exploring the integration of their dual functions on monitoring and removal for heavy metals in both inorganic and organic forms. At the same time, in the process of using metal-based SANs to monitor and control pollutants, the introduction of new heavy metal pollution to environment should be avoided.

## Conclusion and Future Perspectives

Monitoring and controlling the environmental pollutants is a pressing issue concerning global public health. Exploring the advanced technologies for the efficient detection and mitigation of pollutants is crucial in addressing the challenges posed by environmental pollution. As demonstrated by the examples discussed in the previous section, the significant advances have been made in employing SANs-based approaches for environmental monitoring and control. Nonetheless, the practical implementation of SANs is still subject to several challenges:

1. At present, owing to the unclear structure–function relationship of most SANs, the development of most SANs follows the trial-and-error verification approach, and the catalytic activity of developed SANs still needs to be further improved. In future, the computer simulation software could be utilized to model and simulate the structure of SANs, predict their possible functional sites and action mechanism, and then validate these predictions through experiments.
2. Most of the currently reported synthesis methods for SANs are relatively complex, which inevitably leads to certain shortcomings like low metal loading and reduced catalytic efficiency, probably hindering their practical application in environmental remediation. Specifically, it is feasible to synthesize nanomaterials at reduced temperatures by employing low-melting-point precursors, metal–organic chemical vapor deposition (MOCVD), plasma-enhanced chemical vapor deposition (PECVD), etc. [181]. Furthermore, functional single-atom materials can be selectively designed and tailored to meet specific application requirements. For example, optimizing the coordination environment of metal atoms would enhance metal loading and create more active sites, thereby improving catalytic performance [182].
3. Most current reports on SANs for environmental monitoring and control remain at the laboratory stage owing to the presence of the complex background matrices that coexist with the target pollutants in the real environment-related matrices. These matrices may disrupt the structure of SANs and compromise their stability and reliability in practice applications. Consequently, ensuring the overall performance of these materials necessitates addressing how to enhance the stability and long-term dispersion of SANs. This is a critical topic for future research, particularly concerning their practical applications, which may involve selecting the appropriate metal precursors and the substrate materials that exhibit strong interactions [183].
4. The existing literatures on SANs have indicated a limited number of approaches for the integration of detection and degradation processes, which is more in line with the practical demand. Future research should focus on developing the additional integrated SANs-based methods for the simultaneous detection and degradation of environmental pollutants [184].
5. The environmental safety, biocompatibility and toxicity of SANs brings another challenge for their use in practice. With the increasing emphasis on SANs in environmental remediation applications, the feasibility of SANs in real environmental applications should be evaluated in the future to avoid new pollution problems.
6. The integration of artificial intelligence (AI) and nanomaterials synthesis is advancing the frontiers of materials science. For example, Zhang et al*.* developed an AI-driven platform known as Carbon Copilot, which enables the efficient synthesis of carbon nanotubes through high-throughput screening of catalysts coupled with the precise control over growth conditions. This platform not only increased the productivity, but also achieved the breakthroughs in catalyst innovation and density control [185]. Inspired by this, by leveraging AI algorithms alongside automated experimental platforms and efficient data processing techniques, smart synthesis methods can be employed to further screen and design more effective and selective SANs, enhancing their catalytic properties while minimizing the dosage requirements and reducing the side-effects toward environmental health.

With the continuous development of SANs, their application in monitoring and control of environmental pollutants presents a promising opportunity to overcome the limitations associated with traditional methods. We anticipate that this systematic review will offer valuable strategies for researchers aiming to develop innovative, stable, efficient, intelligentized, and cost-effective environmental surveillance, traceability, and remediation techniques. Furthermore, it seeks to facilitate the innovation and progress on the synthesis of robust and effective SANs while enhancing the understanding of the latest developments in their application for monitoring and control of environmental pollutants, ultimately contributing to reduced the risks to environmental health. Finally, we expect the significant progress in the development of novel SANs tailored for real environmental applications concerning the monitoring and control of environmental pollutants, especially for emerging contaminants. Additionally, we foresee that SANs-driven portable smart devices designed for convenient surveillance, traceability, and management of environmental pollutants will be also commercialized in the near future.
